# Profiling the knowledge of female medical/para-medical students, and expertise of health care professionals on female genital schistosomiasis in Anambra, South Eastern Nigeria

**DOI:** 10.1371/journal.pntd.0011132

**Published:** 2023-02-16

**Authors:** Ogechukwu B. Aribodor, Hammed O. Mogaji, Olabanji A. Surakat, Nwadiuto O. Azugo, Eunice C. Jacob, Emmanuel M. Obikwelu, Obiageli J. Nebe, Julie Jacobson

**Affiliations:** 1 Department of Zoology, Nnamdi Azikiwe University, Awka, Nigeria; 2 Parasitology and Epidemiology Unit, Department of Animal and Environmental Biology, Federal University Oye-Ekiti, Nigeria; 3 Department of Zoology, Faculty of Basic and Applied Sciences, Osun State University, Osogbo, Nigeria; 4 Neglected Tropical Diseases Unit, Anambra State Ministry of Health, Awka, Nigeria; 5 Neglected Tropical Diseases Division, Federal Ministry of Health, Nigeria; 6 Bridges to Development, Vashon, Washington, United States of America; Institute of Continuing Medical Education of Ioannina, GREECE

## Abstract

**Background:**

Female genital schistosomiasis (FGS) is a largely neglected tropical disease (NTD), with little or no attention in the primary health care unit. Towards building momentum to address this challenge, we investigated the perception of medical and para-medical students about FGS, as well as the expertise of health care professionals in Anambra State, Nigeria.

**Methodology:**

We conducted a cross-sectional survey among 587 female medical and para-medical university students (MPMS), and 65 health care professionals (HCPs) saddled with the responsibility of delivering care for schistosomiasis-affected persons. Pretested questionnaires were administered to document the awareness and knowledge about the disease. In addition, the expertise of HCPs vis-à-vis suspicion of FGS and management of FGS patients during routine health care service were documented. Data were subjected to descriptive, chi-square tests and regression analysis in R software.

**Results:**

Over half of the students recruited; 54.2% for schistosomiasis and 58.1% for FGS, were not aware of the disease. Knowledge about schistosomiasis was associated with student’s year of study, with those in 2^nd^ (OR: 1.66, 95% CI: 1.0, 2.7), 4^th^ (OR: 1.97, 95% CI: 1.2, 3.2), and 6^th^ (OR: 5.05, 95% CI: 1.2, 34.2) year having higher likelihoods of been more informed about schistosomiasis. For HCPs, we observed a contrastingly high knowledge about schistosomiasis (96.9%), but low knowledge about FGS (61.9%). Knowledge for both schistosomiasis and FGS was not associated with year of practice and expertise (95% OR included 1, p > 0.05). A considerable proportion (>40%) of the HCPs never suspected schistosomiasis during routine clinical diagnosis of patients who presented probable FGS symptoms (p < 0.05). Similarly, only 20% were certain about the use of praziquantel for treating FGS, and about 35% were uncertain of the eligibility criteria and dosage regimens. Commodities for managing FGS were also largely unavailable in about 39% of the health facilities where the HCPs operate.

**Conclusion:**

Awareness and knowledge about FGS among MPMS and HCPs were poor in Anambra, Nigeria. It is therefore important to invest in innovative methods of building capacity of MPMS and HCPs, with complementary provision of necessary diagnostics to perform colposcopy, as well as competence to diagnose pathognomonic lesions using diagnostic atlas or Artificial Intelligence (AI).

## Author summary

Female genital schistosomiasis (FGS) is a neglected disease that is often mistaken for other sexually transmitted infections. The disease has thus received little attention at the primary health care unit until recent. To support the emerging momentum been built towards controlling this disease, we conducted this study to understand the knowledge and perception of FGS among medical and paramedical students (MPMS) who subsequently transform into HCPs, as well as expertise of current HCPs who are saddled with the responsibility of delivering care to FGS patients. 587 MPMS and 65 HCPs were interviewed in Anambra State, using well designed questionnaires. Our findings show that majority of the MPMS were not aware of schistosomiasis and FGS, but students in the 6^th^ year of study have higher chances of been informed about schistosomiasis. However, almost all the HCPs were aware of schistosomiasis, but FGS awareness was very low. The HCPs also lack the capacity and resources to diagnose and manage FGS. This significant gap in knowledge, capacity and resources calls for development of FGS training materials, and its subsequent incorporation in curricula of medical students, and training of HCPs during routine schistosomiasis control programs. This should be complemented with the provision of necessary diagnostics to perform colposcopy, as well as building competence to diagnose pathognomonic lesions using diagnostic atlas or Artificial Intelligence (AI).

## Background

Schistosomiasis is one of the most common neglected tropical diseases (NTD) in the world, with over 206 million people affected [1]. This disease is caused by parasitic water-borne trematodes of the genus *Schistosoma*, and over 90% of those affected reside in Africa. Two major *Schistosoma* species are common in Africa, *S*. *mansoni* and *S*. *haematobium*, with the former and latter causing intestinal and urogenital schistosomiasis, respectively [2]. The pathologies associated with both species vary depending on several factors, which are not limited to the severity of infection (duration and intensity), migration of the worms through the organs and body tissues, and inflammatory responses to the presence of the eggs laid by the adult worms [3]. Intestinal schistosomiasis can result in symptoms such as abdominal pain, diarrhea, blood in the stool, and in more severe cases, enlargement of the liver and spleen, a condition known as hepatosplenomegaly [3,4]. The inflammatory reactions in the liver could lead to hepatosplenic schistosomiasis, which is a key feature of chronic infection. The fibrotic lesions produced by this inflammation could lead to liver cirrhosis that progressively occludes the portal system giving rise to portal hypertension, which could eventually lead to death [5]. Hematuria, which is classified as the passage of visible or invisible blood in urine is a common symptom of urogenital schistosomiasis [3]. Other complicated pathologies of urogenital schistosomiasis may include fibrosis of the bladder and ureter, kidney damage, and in more advanced cases cancer of the bladder [3]. Urogenital schistosomiasis may become more complex in females in a condition known as Female Genital Schistosomiasis (FGS).

FGS is a clinical condition used to describe the presence of trapped *S*. *haematobium* eggs, DNA, with characteristic clinical changes specifically in the genital tract of affected women, regardless of whether or not, the eggs are present in the urinary tract [6,7]. Many women acquire *S*. *haematobium* infection in childhood during domestic and recreational activities at infested water bodies, and about 75% of them may develop FGS [6,8]. Clinical manifestations such as vaginal bleeding, discharge, hematuria, dysuria, dyspareunia, and post-coital bleeding are partly caused by trapped eggs which damage the genital mucosal linings with their characteristic terminal spine, and majorly the inflammatory responses to the embedded eggs [9]. This situation makes those affected more susceptible to HIV and Human Papillomavirus infections [10]. In addition, blockage of the uterus and/or fallopian tubes by the eggs or inflammatory responses to the egg may result in fertility problems. Furthermore, for reproductive women, FGS is associated with poor pregnancy outcome such as stillbirth, spontaneous abortion, and ectopic pregnancy [11]. These reproductive health consequences (fertility issues and poor pregnancy outcome) often lead to discrimination, depression, marital discord and social stigma, particularly when FGS is mistaken for other sexually transmitted infections (STI) [11–13]. In adult females, eggs are commonly trapped in the cervix, vagina, and vulva, while vulvar lesions are common in younger females [6,7,12,14]. FGS lesions may present themselves as grainy sandy patches [7], homogenous yellow patches [6], and rubbery papules.

As of 2020, there has been no assessment of FGS by the Global Burden of Disease study, either as a single entity or as part of the burden of schistosomiasis [15]. However, based on isolated reports, it is estimated that FGS affects about 56 million women and girls in Africa, and about 20 to 150 million females of all ages are estimated to be at risk of infection [16]. Foreigners from non-endemic countries who visit infested freshwater bodies are also at risk [17]. In West Africa, Nigeria bears the highest burden of schistosomiasis, and invariably FGS. However, only two isolated studies in Ogun [18] and Anambra [19] have investigated the prevalence of FGS in Nigeria. These studies were conducted in rural areas with ecological conditions that supports schistosomiasis transmission [18,19]. Interestingly, the preliminary findings from both states have spurred the interest of healthcare professionals and researchers to understand the epidemiology of FGS and also develop protocols aimed at investigating FGS endemicity in other regions in the country. This interest has been accompanied by investment in capacity building of health care professionals from Bridges to Development, a nonprofit and tax-exempt organization in Switzerland and the United State of America, focused on advancing economic and social well-being of women and girls [20]. Currently, FGS is largely neglected in the training curricula of medical and para-medical university students (MPMS) who subsequently transform into health care professionals (HCPs). These training gaps might contribute to misdiagnosis/classification of clinical manifestations due to FGS, resulting in stigmatization, mental stress, and social exclusion of young females [11–13]. We therefore aim to understand the knowledge and perception of FGS among medical and paramedical students who subsequently transform into HCPs, as well as expertise of current HCPs who are saddled with the responsibility of delivering care to FGS patients. These findings would be useful to support the call for local investments, collaborations and innovations, when deliberations towards controlling FGS are been mapped out by schistosomiasis control programmers, researchers and policy makers in the state, and Nigeria at large.

## Methodology

### Ethics statement and considerations

This study received ethical approval (COOUTH/CMAC/ETH.C/Vol.1/FN:04/0117) from the ethical review board of Chukwuemeka Odumegwu Ojukwu University Teaching Hospital. Consent was sought from study participants prior to data collection, and only participants who provided written informed consent were recruited. Unique identifiers were used in place of participants’ name to ensure anonymity, while a password-protected database was used to archive all datasets to ensure confidentiality.

### Study area

**Fig 1:**
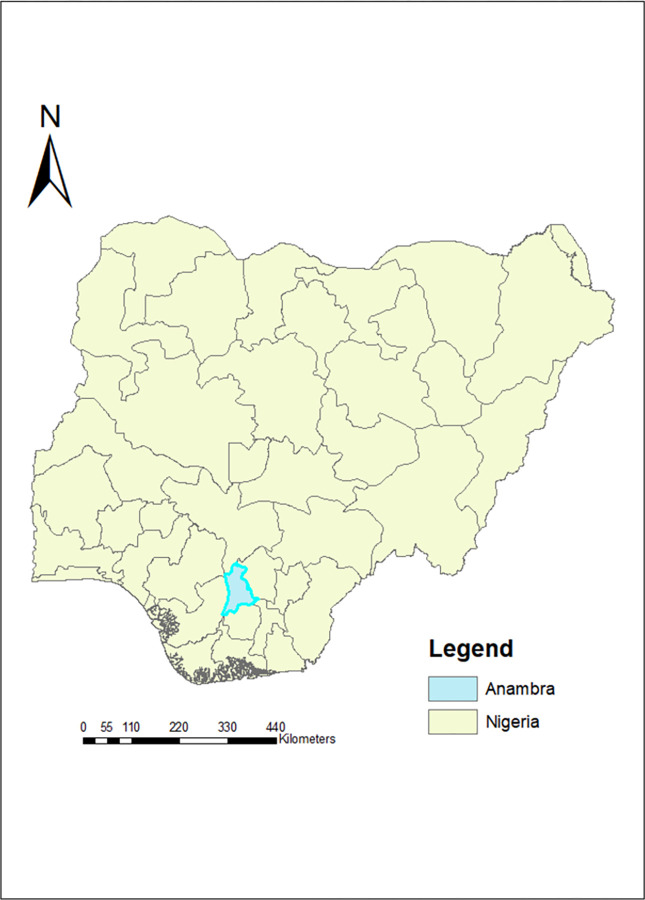
Map of Nigeria showing Anambra State. Source: The authors created this map in ArcGIS 9.3 software. The basemap shapefiles of the 37 administrative units in Nigeria unto which we plotted our map were downloaded from an open and publicly accessible database of openAFRICA at https://africaopendata.org/dataset/shape-file-of-nigeria/resource/372a616a-66cc-41f7-ac91-d8af8f23bc2b.

Anambra is one of the 36 states in Nigeria, located in the southeastern part of the country, with 21 administrative regions known as local government areas (LGAs) (Fig 1). By population estimate, Anambra is ranked the 14^th^ most populated state [21], with a very low rurality index following Lagos [22]. However, the presence of arable soil, tropical rain forest, topography, lakes, and river favors both farming/ fishing activities, as well as the transmission of schistosomiasis [23,24]. In 2017, the most recent date in which statistics are available, there were over 590 public and 1600 private healthcare facilities operating in Anambra state [25], however, there are concerns that healthcare centers in rural areas lack trained healthcare personnel [26].

### Study design, mobilization, recruitment of study participants, and data collection

This study was conceptualized following the guidelines of Bridges to Development, and The Geneva Learning Foundation on the FGS Accelerated Scale Together (FAST) Package. The study was designed as a cross-sectional survey to assess the perception and expertise of female MPMS and HCPs using quantitative methods. Two electronic questionnaires were developed during the First Scholar Workshop on Female Genital Schistosomiasis (FGS) (https://www.learning.foundation/female-genital-schistosomiasis) and the First FGS Impact Accelerator meeting (https://www.learning.foundation/fgs) held between May and October 2021. Questionnaires were pilot tested among two external supervisors and 10 female students in Anambra, before deploying it for field use in October 2021. However, physical interactions were impossible in October, because of COVID-19 lockdown orders and closure of schools during the strike actions by academic unions across all government -owned universities in the country. As such, we employed electronic mode of enrollment and administration of questionnaires. We therefore recruited medical and para-medical female students enrolled in Nnamdi Azikiwe University in Awka. This university was purposively selected because it is government-owned, and with the largest enrollment due to its low tuition fee when compared to other universities in the state. Also, the rationale to include only female students in our KAP assessment was borne out of the interest to explore experiences of females with probable symptoms of FGS when they sought medical care. This rationale is flawed considering the fact that awareness and knowledge about FGS should be of concern to both gender. We have thus described this as one of the limitations of the study.

A minimum of 500 medical and paramedical female students from the three faculties; biosciences, medical sciences and pharmacy, were targeted for recruitment. Students enrolled in the faculty of medicines and pharmacy were grouped as medical students, while those enrolled in biomedical courses in the faculty of biosciences were grouped as paramedical students. These students were approached through the headship of faculty-based student unions, and subsequently invited to participate in the study through personal invitation letters sent to their institutional email. Follow-up recruitments were done through dedicated WhatsApp and Telegram platforms. An electronic packet that contains a brief introductory note about the project, a question to seek consent, and the main questionnaire were sent across the platforms. Only submissions of those who completed the consent questionnaire were included in the final analysis.

Furthermore, we targeted 50 of the 100 health care practitioners who operate at the State level, and are members of the schistosomiasis control committee. These healthcare practitioners (HCPs) were interviewed physically during one of the routine stakeholder meetings organized by the Anambra State Schistosomiasis control and elimination program. Questionnaires were self-administered to consenting stakeholders before the commencement of the meeting. Considering the literacy status of participants (MPMS and HCPs), all questionnaires were designed and administered in English language.

### Study indicators

We used two separate tools (See https://tinyurl.com/mkudxmsw) and (See https://tinyurl.com/mudvvh7t) to document responses from female MPMS and HCPs, respectively. For HCPs, demographic information including age, gender, profession, year of practice and place of work were documented. In addition, we collected information on HCPs expertise on a range of symptoms/condition that overlaps with FGS pathology. These included; (i) if the practitioner performs pelvic examination, (ii) treat venereal diseases, (ii) lower abdominal pain, (iv) dysuria or (v) hematuria. Knowledge of HCPs about schistosomiasis and FGS were also documented, with emphasis on (i) awareness, (ii) source of information, (iii) transmission routes, (iv) preventive practices, (v) treatment choices with emphasis on pregnant women, and (vi) dosage requirements for children and adult. HCPs were also asked if FGS patients should be quarantined and if care commodities for FGS patients are available in their place of work (See S1 File)

Similarly for female MPMS, we collected information on age, religion, parents’ occupation and income. In addition, knowledge about schistosomiasis and FGS were documented, with emphasis on awareness and source of information. We also documented if MPMS (i) have ever been treated with praziquantel, (ii) had contact with freshwater, or (iii) had experienced any of the following symptoms that overlaps with FGS pathology; hematuria, painful urination, lower abdominal pain and venereal diseases (See S1 File).

### Data management and analysis

Data collected (See S1 File) were imported from password protected online repository into R studio for analysis. Descriptive statistics such as frequencies and percentages were used to summarize; (1) knowledge and awareness of schistosomiasis and FGS among students and HCPs, (2) prevalence of schistosomiasis suspicion among practitioners who give FGS care, (3) availability of FGS care commodities at health care centers, and (4) students’ experience when they sought care on FGS-related symptoms. Chi-square tests was used to examine differences between proportion, and a univariate logistic regression model was performed to estimate if the year of study for the medical and paramedical students influences their knowledge about schistosomiasis and FGS. Additionally, we extracted the anonymized dataset of students who had experienced some of the probable symptoms of FGS and had visited the hospital for medical care. We performed a cross-tabulation analysis on two variables; (i) the symptoms experienced, and (ii) if the practitioner who provided care asked about a major risk factor for FGS (i.e., freshwater contact practice). Chi-square test was also used to test for associations. Similarly for the HCPs, we modelled knowledge about schistosomiasis and FGS against year of practice (less or greater than 10 years) and expertise in performing pelvic examinations; treating venereal diseases; hematuria, lower abdominal pain, dysuria, or and previous suspicion of schistosomiasis during such examinations/treatment. Odd ratios (OR) and 95% Confidence Intervals (CI) were reported accordingly, with significant level established at p < 0.05. All statistical procedures were performed in R studio.

## Result

### Demographic characteristics of the study participants

Table 1 shows the demographic characteristics of the participants. A total of 587 female medical and para-medical students and 65 healthcare professionals participated in this study. The majority of the female students (59%) were within the age category 20–24 years, followed by those between the age category 15 and 19 years. Majority of the students were also within in their 1^st^ (25%), 2^nd^ (22.5%), 3^rd^ (19.3%) and 4^th^ (24%) year of medical school. About 74% of them were from households that earn above the minimum national income of $50 per month. On the other hand, about 52% of the healthcare professionals recruited were females and 48% were males. About 56% of them were within the age category of 25–45 years. Eight different fields of medical practice were represented, with a majority of the practitioners being medical doctors (36.9%), followed by laboratory scientists (21.5%), and community health workers (10.8%). Other specialization included researcher, nurse, pharmacist, public health officer, and radiographer. About half of the practitioners have spent less than a decade in their current position. Also, 87% of the practitioners work in a government-owned facility. However, only 46% of them have experience conducting pelvic examinations.

**Table 1:** Demographic characteristics of the study participants.

	Frequency	%
**Female medical and paramedical students (n = 587)**		
**Age category (in years)**		
15–19	182	31
20–24	345	58.8
25–29	56	9.5
>30	4	0.7
Year of study		
1^st^ year	147	25.0
2^nd^ year	132	22.5
3^rd^ year	113	19.3
4^th^ year	141	24.0
5^th^ year	33	5.6
6^th^ year	10	1.7
7^th^ year	11	1.9
**Average household income**		
<50$	148	25.2
>50$	433	73.8
Undefined	6	1.0
**Healthcare professionals (n = 65)**		
**Gender**		
Male	31	47.7
Female	34	52.3
**Age category (in years)**		
20–24	7	10.8
25–29	14	21.5
30–34	7	10.8
35–39	7	10.8
40–44	11	16.9
45–49	7	10.8
>50	12	18.5
**Years of practice**		
<10	34	52.3
11–20	15	23.1
21–30	10	15.4
31–40	1	1.5
41–50	1	1.5
No response	4	6.2
**Specialization**		
Researcher [Academic]	3	4.6
Researcher [Clinical]	2	3.1
Community Health Worker	7	10.8
Laboratory Scientist	14	21.5
Medical Doctor	24	36.9
Nurse	5	7.6
Pharmacist	4	6.2
Public health officer	5	7.7
Radiographer	1	1.5
**Type of establishment**		
Government-owned	57	87.7
Private	8	12.3
**Conduct pelvic examinations?**		
**Yes**	30	46.2
**No**	35	53.8

### Awareness about schistosomiasis and FGS among female students

**Fig 2:**
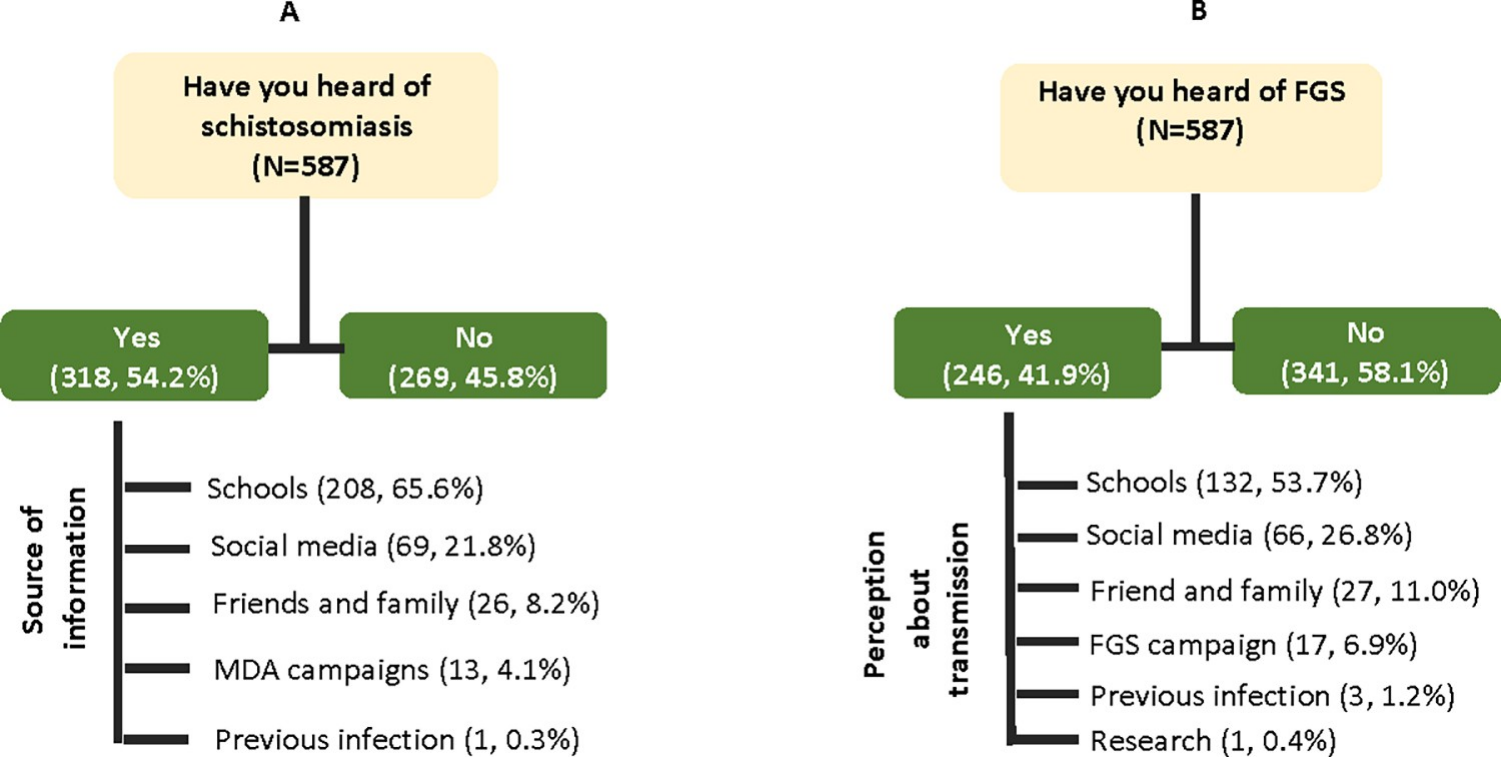
Awareness of schistosomiasis and FGS among university students.

Table 2 show the awareness about schistosomiasis and FGS among female students. Of the 587 students interviewed, about half of them 318 (54.2%) affirmed they have heard of schistosomiasis. A majority reported their source of information was from the school environment 208 (65.6%), followed by social media 69 (21.8%), friends and family 26(8.2%), and through schistosomiasis mass drug administration (MDA) campaigns 13 (4.1%) (Fig 2). However, only 246 (41.9%) of the participants have heard about FGS. (Table 2) Among those that have heard, their source of information was from the school environment 132 (53.7%), followed by social media 66 (26.8%), friend and family 27 (11%) and through female genital schistosomiasis campaigns 17 (6.9%).

**Table 2:** Regression analysis between knowledge of study participants, students’ year of study, and expertise of health care professionals.

		Have you heard of Female Genital Schistosomiasis				Have you heard of Schistosomiasis			
	NE	No	Yes	OR (95%CI)	p	No	Yes	OR (95% CI)	p
**Medical/para-medical students**									
**Level of study**									
1^st^ year	147	87 (59.2)	60(40.8)	1	-	82(55.8)	65(44.2)	1	-
2^nd^ year	132	79 (59.8)	53(40.2)	0.97(0.6,1.6)	0.91	57(43.2)	75(56.8)	1.66(1.0,2.7)	0.04*
3^rd^ year	113	68 (60.2)	45(39.8)	0.96(0.6,1.6)	0.87	53(46.9)	60(53.1)	1.43(0.9,2.3)	0.16
4^th^ year	141	80 ((56.7)	61(43.3)	1.11(0.7,1.8)	0.67	55(39.0)	86(70)	1.97(1.2,3.2)	0.00*
5^th^ year	33	17 (51.5)	16(48.5)	1.36(0.6,2.9)	0.42	15(45.5)	18(54.5)	1.51(0.7,3.3)	0.28
6^th^ year	10	4 (40)	6(60)	2.17(0.6,8.8)	0.24	2(20)	8(80)	5.05(1.2,34.2)	0.05*
7^th^ year	11	6 (54.5)	5(45.5)	1.21(0.3,4.2)	0.76	5(45.5)	6(54.5)	1.51(0.4,5.5)	0.51
Total	587	341(58.1)	246(41.9)			269(45.8)	318(54.2)		
p-value			0.865				0.071		
**Health care professionals (HCPs)**									
**Year of practice**									
<10 years	34	11(32.4)	23(67.6)	1		0(0)	34(100)	-	-
Above 10 years	31	14(45.2)	17(54.8)	0.58(0.2,1.6)	0.29	2(6.5)	29(93.5)	-	-
Total	65	25(38.1)	40(61.9)			2(3.1)	63(96.9)		
p-value			0.421			0.432			
**Perform pelvic examinations**									
No	35	16 (45.7_	19(54.3)	1	-	1(2.9)	34(97.1)	1	-
Yes	30	9 (30)	21(70.0)	1.96(0.7,5.6)	0.2	1(3.3)	29(96.7)	0.85(0.3,22.2)	0.91
Total	65	25(38.1)	40(61.9)			2(3.1)	63(96.9)		
p-value			0.2972			1.00			
**Do you treat patients with;**									
**A: venereal diseases**									
No	24	8(33.3)	16(66.7)	1		1(4.2)	23(95.8)	1	
Yes	41	17(41.5)	24(58.5)	0.71(0.2, 1.9)	0.52	1(2.4)	40(97.6)	1.74(0.07,45.4)	0.7
Total	65	25(38.1)	40(61.9)			2(3.1)	63(96.9)		
p-value		0.699				1.000			
**B: lower abdominal pain**									
No	24	10(41.7)	14(58.3)	1		1(4.2)	23(95.8)	1	
Yes	41	15(36.6)	26(63.4)	1.24(0.4,3.5)	0.68	1(2.4)	40(97.6)	1.74(0.07,45.4)	0.7
Total	65	25(38.1)	40(61.9)			2(3.1)	63(96.9)		
p-value		0.887				1.000			
**C: hematuria**									
No	24	9(37.5)	15(62.5)	1	0.9	1(4.2)	23(95.8)	1	
Yes	41	16(39.0)	25(61)	0.94(0.3, 2.6)		1(2.4)	40(97.6)	1.74(0.07,45.4)	0.7
Total	65	25(38.1)	40(61.9)			2(3.1)	63(96.9)		
p-value		1.000				1.000			
**D: dysuria**									
No	32	14(43.8)	18(56.2)	1		2(6.3)	30(93.7)	-	-
Yes	33	11(33.3)	22(66.7)	1.56(0.6,4.3)	0.39	0(0)	33(100)	-	-
Total	65	25(38.1)	40(61.9)			2(3.1)	63(96.9)		
p-value		0.543				0.459			
**Do you suspect schistosomiasis when treating any of (A-D)**									
No	27	14(51.9)	13(48.1)	1		2(7.4)	25(92.6)	-	-
Yes	29	8(27.6)	21(72.4)	2.83(0.95,8.91)	0.07	0(0)	29(100)	-	-
Total	65	25(38.1)	40(61.9)			2(3.1)	63(96.9)		
p-value		0.113				0.440			

NE: Number examined; OR: Odd ratio; CI: Confidence interval; p: p-value; *p is significant at 0.05

### Knowledge of health care professionals about schistosomiasis and FGS

**Fig 3:**
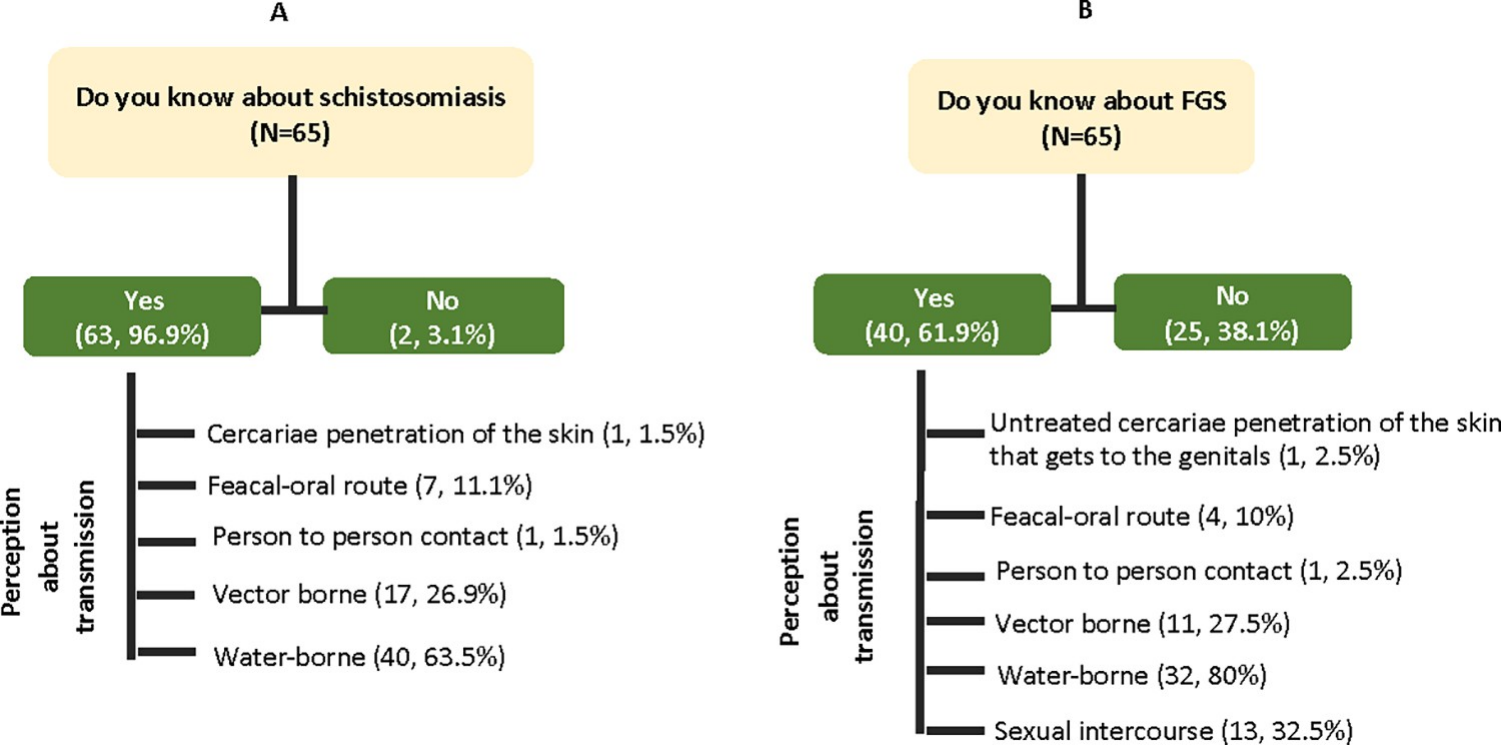
Knowledge of health care professionals about schistosomiasis and FGS.

Table 2 also show the knowledge of HCPs about schistosomiasis and FGS among female students. Of the 65 practitioners interviewed, 63 (96.9%) of them affirmed they have heard of schistosomiasis. The majority of these respondents 40 (63.5%) explained that it is a water-borne disease, followed by 17 (26.9%), who claimed it is transmitted by vectors. Other respondents claimed it can be transmitted through the feacal-oral pathway, 7 (11.1%) and person-to-person contact, (1, 1.5%). Only one respondent reported that it is caused by cercariae penetrating the skin of a susceptible host (Fig 3). However, when asked about FGS, only 40 (61.9%) of the participants know about the disease. The majority of these respondents 32 (80%) explained that it is a water-borne disease, followed by sexual intercourse (13, 32.5%) and 11 (27.5%), who claimed it is a disease transmitted by vectors. Other respondents claimed it can be transmitted through the feacal-oral pathway, 4 (10%) and person-to-person contact, (1, 2.5%). Only one respondent reported that it is caused by the cercariae penetrating the skin of a susceptible host which later gets to the genitals (Fig 3).

### Regression analysis between knowledge of study participants, students’ year of study, and expertise of health care professionals

Table 2 also show the results of regression analysis performed between knowledge of study participants, students’ year of study, and expertise of health care professionals. There were significant association between the students’ year of study and awareness about schistosomiasis. Students in the 2^nd^ (OR: 1.66, 95% CI: 1.0, 2.7), 4^th^ (OR: 1.97, 95% CI: 1.2, 3.2), and 6^th^ (OR: 5.05, 95% CI: 0.4, 5.5) year have higher chances of been more informed about schistosomiasis than others. However, there were no significant association between year of study and knowledge about FGS (95% CI included 1 and p > 0.05). (Table 2).

Furthermore, of the 65 practitioners interviewed, about 45% had spent more than 10 years in practice. By expertise, only 30(46.2%) of them perform pelvic examinations while 41 (63.1%) of them provide patient-based care to people with venereal diseases, significant lower abdominal pain, and hematuria, respectively. However, 33 (50.8%) of them provide care to patients presenting dysuria, and 29(44.6%) reported they had suspected schistosomiasis during routine care for people with venereal diseases, significant lower abdominal pain, and hematuria (Table 2). The regression analysis between knowledge, year of practice and expertise of health care professionals showed no significant association (95% CI included 1 and p > 0.05). (Table 2).

### Prevalence of schistosomiasis suspicion among practitioners during routine practice

Table 3 show the prevalence of schistosomiasis suspicion among practitioners who offer care. Majority of the practitioners suspected schistosomiasis during their diagnosis of dysuria (60.6%), hematuria (58.5%), significant lower abdominal pain (56.1%) and venereal diseases (53.7%). Almost 90% of the practitioners who provide care for patients with venereal diseases have used praziquantel as a drug of choice when they suspect schistosomiasis. There were significant differences in the proportions reported for schistosomiasis suspicion across the different types of conditions treated (p < 0.05) (Table 3).

**Table 3:** Prevalence of schistosomiasis suspicion among practitioners during routine practice.

		Suspect a possible schistosomiasis infection in your diagnosis			p-value
**Practitioners who treat**	NT	Yes	No	NA	
Venereal diseases	41(63.1)	22(53.7)	18(43.9)	1(2.4)	0.004
Significant lower abdominal pain	41(63.1)	23(56.1)	17(41.5)	1(2.4)	0.002
Hematuria	41(63.1)	24(58.5)	16(39.1)	1(2.4)	0.007
Dysuria	33(50.8)	20(60.6)	12(36.4)	1(3.0)	0.010
Use praziquantel to treat venereal diseases	24(36.9)	21(87.5)	3(12.5)	0(0)	0.000

NT: Number of practitioners who treat one form of clinical condition; NA: Not applicable; p-value is significant at 0.05

### Management of FGS patients during routine health care service

**Fig 4:**
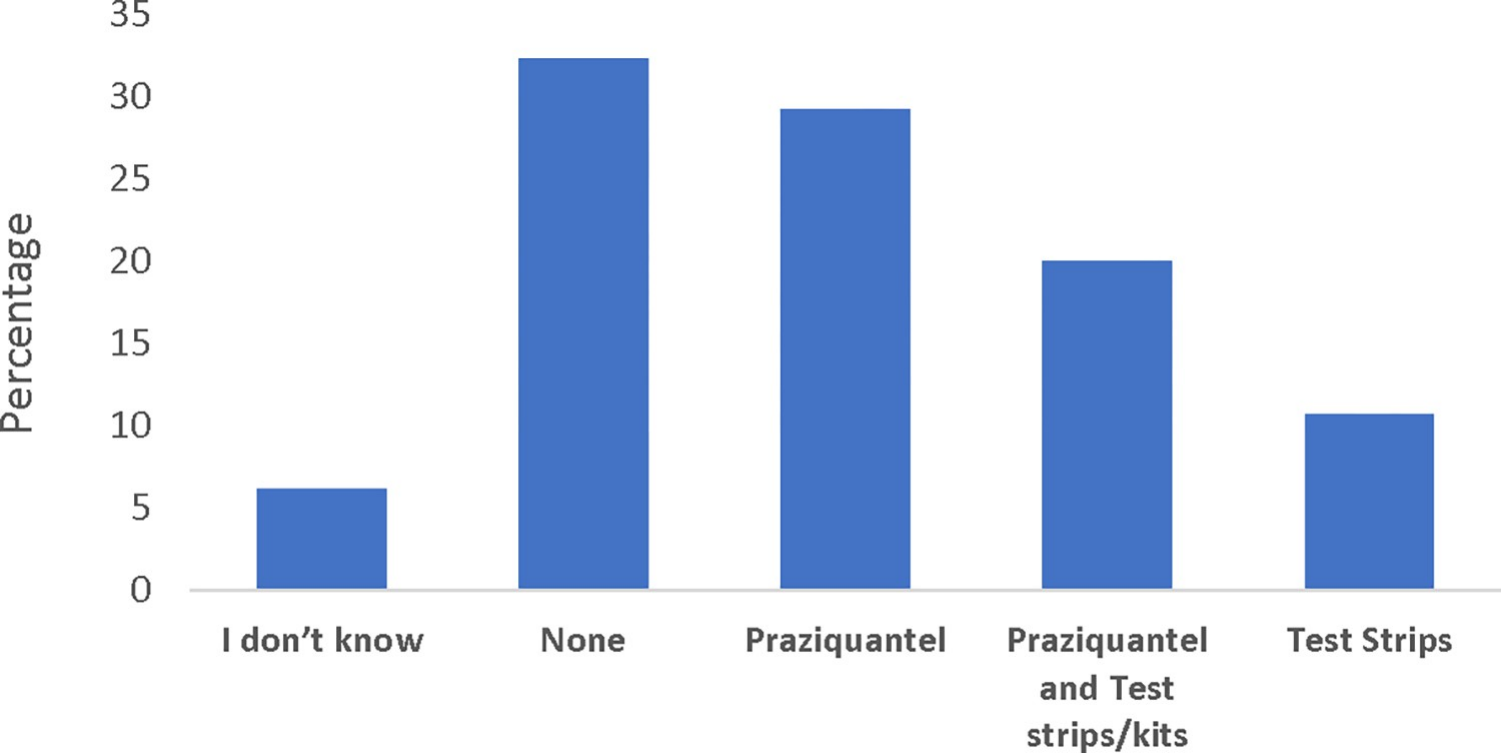
Availability of commodities at health care centers for FGS patients.

About 43% of the practitioners were uncertain if they should quarantine patients with FGS. Similarly, only 20% of the practitioners affirmed that praziquantel is the only drug of choice for treating FGS (Table 4). A considerable proportion (29.3%) of them were uncertain if they were to treat pregnant women, and about 25% wrongly affirmed they could treat during any of the trimester of pregnancy. In addition, about 35% of them do not know the dosage requirements for praziquantel tablets (Table 3). Fig 4 shows availability of commodities across the health care centers where HCPs operate. Commodities such as medications and equipment for managing FGS were largely unavailable (39%) in the workplaces of the practitioners. However, about 29% reported having access to praziquantel in their workplace, while 20% reported having both praziquantel and schistosomiasis detection kits or urinalysis test strips and 11% reported having only test strips (Fig 4).

**Table 4:** Knowledge about the management of FGS patients during routine healthcare service.

N = 41	Frequency	%
**Persons affected with FGS should be quarantined.**		
No	23	56.1
Don’t know	13	31.7
Maybe	5	12.2
**Praziquantel is the only treatment for FGS.**		
Yes	8	19.5
No	17	41.5
I don’t know	16	39.0
**Praziquantel can be used to treat pregnant women.**		
Yes	29	70.7
No	12	29.3
**In which trimester can pregnant women take praziquantel?**		
First	0	0
Second	9	21.9
Third	18	43.9
All	10	24.4
None	4	9.8
**The praziquantel dosage is the same for adults and children**		
Yes	2	4.9
No	26	63.4
I don’t know	13	31.7

### Participants’ experiences during routine healthcare service on FGS-related symptoms

Of the 587 female MPMS, 83 presented a case of painful urination to the health care center, and only 22 (26.5%) of them were asked if they had recent contact with fresh water bodies (p = 0.458). Similarly, only 12 (38.7%) of the 31 students who presented hematuria were asked (p = 0.035). In addition, only 44 (23.5%) of 187 who presented lower abdominal pain were asked (p = 0.786), and only 31 (37.3%) of the 83 residents who presented symptoms of venereal diseases were asked (p = 0.000) (Table 5).

**Table 5:** Participants’ experiences during routine health care service on FGS-related symptoms.

	The practitioner asked about recent contact with freshwater			
**Participant’s symptom**	NE	Yes	No	p-value
Painful urination	83	22 (26.5)	61 (73.5)	0.458
Hematuria	31	12 (38.7)	19 (61.3)	0.035
Lower abdominal pain	187	44 (23.5)	143 (76.5)	0.786
Venereal diseases	83	31 (37.3)	52 (62.7)	0.000

NE: Number examined; p-value is significant at 0.05

## Discussion

Schistosomiasis has been largely studied in Nigeria [27,28], and a network of partnerships between national and international agencies has invested commendable resources to control the menace of the disease [29,30]. In 2019, about 250 million praziquantel tablets were administered in endemic communities, with the sole beneficiaries being children under age 15 [1]. Until recent, adults, most especially females of reproductive age (>15 years) have been largely neglected in schistosomiasis control programs [31], owing to the premise that mass administration of praziquantel to children under 15 is sufficient to reduce the burden of schistosomiasis in an endemic setting [32]. Unfortunately, women living in endemic regions could acquire *S*. *haematobium* infection in childhood [19,33,34], which may develop into female genital schistosomiasis (FGS) when left untreated [6,8,18,31]. The clinical manifestations and consequences of FGS, on both the sexual and reproductive health of females, have been extensively described [9], and in recent times have gained public health traction [35].

However, despite the fact that FGS affects about 56 million women and girls in Africa, with about 20 to 150 million females estimated to be at risk, the disease remains largely neglected [16]. This is partly because the clinical manifestations are often misdiagnosed and confused with those of other sexually transmitted infections (STIs) [13], which in most cases do not only lead to undertreatment of genuine FGS cases but also stigmatization, mental stress, social exclusion and impaired life quality among young females who were wrongly misdiagnosed to have STIs [13,15,36]. Efforts targeted at addressing this capacity gap, most especially among healthcare professionals are emerging, for example with the FAST package implemented in Ghana and Madagascar [37] and other capacity-building workshops organized by Bridges to Development [38]. In this study, we, therefore, characterized the profiles of the university student vis-a-vis their knowledge and awareness about FGS, since they are most likely to take on healthcare-related jobs in the subsequent future. In addition, we assessed the expertise and challenges of healthcare professionals that are currently engaged in schistosomiasis control activities as a precursor to developing interventions to support the control of FGS.

Firstly, our findings revealed poor knowledge and awareness about schistosomiasis and FGS among medical and paramedical students, with majority of those that have heard about the disease attributing the source of information to the school environment and social media. Our regression model found that medical and paramedical students in the second, fourth and sixth year of study have higher odds of been more informed about schistosomiasis, but we couldn’t find any association with FGS. Also, the year of practice and expertise of HCPs was not associated with knowledge of either disease. Due to data limitations, this present study couldn’t explore other factors that are associated with knowledge among study participants. However, our findings reiterate the need to consider investments and innovations in building capacity of medical/para-medical students and HCPs on FGS [35]. In Nigeria, schistosomiasis is taught as part of the course content to university students enrolled in medical and biomedical related disciplines, usually in basic biology classes during their first year, and in a more advanced parasitology course in their third year. However, course synopsis varied across universities and the extent to which regularized contents are been taught cannot be ascertained. The potential of social media and radio programmes in promoting health-educational messages to a wider audience have been reported elsewhere [39,40] and can be explored in this context to promote FGS. Similarly, regulated in-depth training using Massive Open Online Course (MOOC) model can be developed for FGS. This model was recently used by WHO/TDR for building capacity of health researchers and practitioners in implementation research and has contributed significantly to capacity development of 3,858 personnel in 2020 across 115 countries, with about 70% certification rate [41].

We also observed a contrastingly high awareness of schistosomiasis, but low awareness of FGS among healthcare professionals which is in line with previous reports [13,42]. A considerable proportion of our respondents attributed the cause of FGS to contact with water bodies and sexual intercourse, while only a few (2.5%) claimed it is a result of untreated *Schistosoma* infection. The misconception that FGS is transmitted via sexual intercourse needs to be addressed as it could have far-reaching implications on the socioeconomic life of affected women. It should therefore be advocated that FGS is a consequence of embedded *S*. *haematobium* eggs or DNA in the genital tract of affected women [6,7], and the condition is directly related to exposure to infected water bodies harboring the *Bulinus* spp. More importantly, about 40% of the health care professionals claimed they had never suspected FGS among patients who reported symptoms in line with venereal infection. Largely, 60–70% of the female students who reported painful urination, hematuria, lower abdominal pain, and venereal diseases also affirmed that healthcare professionals had never asked them about their history of contact with water-bodies (rivers, lakes and streams) when they sought medical care. The management of FGS was poor among the health care professionals, as a considerable proportion do not know the drug of choice for FGS, and the dosage requirement for treating adults, children, and pregnant women. This lack of awareness about FGS among healthcare workers has been reported elsewhere [13,18,42]. Furthermore, the lack of commodities such as Kato Katz and urine filtration kits which are important diagnostic materials for urogenital and intestinal schistosomiasis is also of concern, as urogenital schistosomiasis is often associated with FGS. This is further worsened by the lack of materials for colposcopy and competence to identify pathognomonic lesions which are necessary tools for diagnosing FGS. It is therefore important to complement discussion around increasing awareness and knowledge of health care professionals, with considerable investments and provision of adequate commodities for detecting and treating schistosomiasis and FGS.

Lack of knowledge and expertise, among health care providers to appropriately diagnose FGS could lead to under or misdiagnosis of the clinical conditions, hence, limiting access to treatment for women and girls suffering from this preventable and treatable disease. It is therefore important to consider interventions targeted at improving healthcare workers’ knowledge by incorporating FGS-related material into medical and para-medical training curricula [13] or more routinely into ongoing schistosomiasis control programme which are implemented at sub-district levels in endemic countries. The latter approach would allow wider coverage to the already engaged local health workforce within implementation units where the disease is most endemic. As previously highlighted by Jacobson [13], this step would require identifying a clear and standardized set of learning outcomes; specifically, the competencies or behaviors required to adequately prevent, diagnose, and manage an FGS case. It is, therefore, important to highlight that currently developed tools can be adapted for local pilot among healthcare professionals in the study area, to improve health workforce capacity and contribute to alleviating the neglect of FGS. This training should also be complemented with provision of appropriate diagnostics and on-site competence building for qualified health care professionals on colposcopy, as well as diagnosis of pathognomonic lesions using diagnostic atlas or Artificial Intelligence (AI) applications [43]

### Conclusion

Our study has highlighted the low level of awareness about FGS among medical/para-medical students and healthcare professionals, as well as poor expertise of healthcare professionals towards FGS care. Our findings show the probable effect of these gaps on underdiagnosis, misdiagnosis, and/or lack of or inappropriate treatment for FGS patients. It is therefore important to develop and incorporate FGS training materials in training curricula of university students and training programs of health professionals during routine ongoing schistosomiasis control programs. Furthermore, this training should be complemented with provision of appropriate medical diagnostics for schistosomiasis and FGS, and on-site competence building on colposcopy, and diagnosis of pathognomonic lesions using diagnostic atlas or Artificial Intelligence (AI) applications.

### Limitations of the study

This study has several limitations; foremost, we only recruited female medical and paramedical students in the survey, which is a major concern as there is a significant lack of information on KAP for FGS among male students. The rationale for this decision during the conceptualization of the study was related to the interest of exploring experiences of females with probable symptoms of FGS when they sought medical care, which we presume would also provide supportive evidence to the assessment of expertise among health care professional. This rationale is flawed, and subsequent studies on KAP for FGS should consider inclusion of both gender in their study design. Secondly, this study was conducted during the COVID-19 pandemic with considerable restrictions on movements, hence we employed an electronic mode of data collection from the recruited medical/para-medical students. In addition, we assessed participants’ awareness/knowledge about schistosomiasis and FGS using a binary outcome [Yes /No], hence we cannot ascertain if respondents know the true definitions and conditions of the diseases. Thirdly, some of the medical and para-medical students affirmed they learnt about schistosomiasis from school, and our quantitative methods could not probe further the exact source of knowledge. We therefore understand their responses may be subject to recall bias. Finally, we recruited a very small number of HCPs (n = 65) who operate at the State level in the schistosomiasis control program, and some of the specializations were underrepresented. We believe a more robust evaluation of practitioner’s expertise at regional and community level may be very useful.

## Supporting information

S1 File Dataset. (XLS)
