# Supplementary material for: Profiling the knowledge of female medical/para-medical students, and expertise of health care professionals on female genital schistosomiasis in Anambra, South Eastern Nigeria
Source: PLoS Negl Trop Dis. 2023 Feb 16;17(2):e0011132. doi: 10.1371/journal.pntd.0011132 (PMC9977039; doi:10.1371/journal.pntd.0011132)
Supplement: Attachment — Submitted filename: PNTD-D-22-01326_reviewer.pdf [file pntd.0011132.s003.pdf]

PLOS Neglected Tropical Diseases

Profiling the knowledge of university female students and expertise of health care professionals in the management of female genital schistosomiasis in Anambra, South Eastern Nigeria.

--Manuscript Draft--

**Manuscript Number:**	PNTD-D-22-01326
**Full Title:**	Profiling the knowledge of university female students and expertise of health care professionals in the management of female genital schistosomiasis in Anambra, South Eastern Nigeria.
**Short Title:**	KAP on schistosomiasis and FGS among students and health care providers
**Article Type:**	Research Article
**Keywords:**	Female Genital Schistosomiasis, Nigeria, Capacity Building, Neglected Tropical Diseases, Health care workers
**Abstract:**	Background<br><br>Female genital schistosomiasis (FGS) is a largely neglected tropical disease, with little or no attention in the primary health care unit. Toward building momentum to address this challenge, we investigated the perception of university female students about FGS, as well as the expertise of health care professionals in Anambra State, Nigeria.<br><br>Methodology<br><br>We conducted a cross-sectional survey among 587 university female students and 65 health care professionals (HCPs) saddled with the responsibility of delivering care for schistosomiasis-affected persons. Pretested questionnaires were administered to document the awareness and knowledge about the disease. In addition, the expertise of HCPs vis-à-vis suspicion of FGS and management of FGS patients during routine health care service were documented. Data were analyzed in SPSS 20.0, and the confidence interval was set at 95%.<br><br>Results<br><br>~~Over half of the students recruited; 54.2% for schistosomiasis and 58.1% for FGS, were not aware of the disease. However, for HCPs, contrastingly high awareness about schistosomiasis (96.9%), but low awareness about FGS (61.9%) was observed.~~ Knowledge about the transmission route for the disease was significantly poor among HCPs. A considerable proportion (>40%) of the HCPs never suspected schistosomiasis during routine clinical diagnosis of patients who presented probable FGS symptoms ($p < 0.05$). Similarly, only 28% were certain about the use of praziquantel for treating FGS cases, and about 35%-39% were uncertain of the eligibility criteria and dosage regimens. Commodities for managing FGS were also largely unavailable in about 39% of the health facilities where the HCPs work.<br><br>Conclusion<br><br>Our study has highlighted the significant lack of awareness and knowledge about FGS among university female students and HCPs in Anambra, Nigeria, and the resultant effect on under/misdiagnosis, and lack of treatment for FGS patients. It is therefore important to develop and incorporate FGS training materials in curricula of medical and para-medical university students, and training programs for HCPs during routine schistosomiasis control programs which has a wider coverage of engaged local health workforce.
**Additional Information:**	
**Question**	**Response**
**Government Employee**	No - No authors are employees of the U.S. government.

Powered by Editorial Manager® and ProduXion Manager® from Aries Systems Corporation

Are you or any of the contributing authors an employee of the United States government?<br><br>Manuscripts authored by one or more US Government employees are not copyrighted, but are licensed under a CC0 Public Domain Dedication, which allows unlimited distribution and reuse of the article for any lawful purpose. This is a legal requirement for US Government employees.<br><br>This will be typeset if the manuscript is accepted for publication.	
**Financial Disclosure**<br><br>Enter a financial disclosure statement that describes the sources of funding for the work included in this submission and the role the funder(s) played. This includes grants and any commercial funding of the work or authors.<br><br>This statement will be typeset if the manuscript is accepted for publication.<br><br>Review the submission guidelines and the instructions link below for detailed requirements and guidance.	Not applicable
**Competing Interests**<br><br>On behalf of all authors, disclose any competing interests that could be perceived to bias this work.<br><br>This statement will be typeset if the manuscript is accepted for publication.<br><br>Review the instructions link below and PLOS NTDs' competing interests policy to determine what information must be	The authors declare that they have no competing interest

disclosed at submission.	
**Data Availability**<br><br>Provide a **Data Availability Statement** in the box below. This statement should detail where the data used in this submission can be accessed. This statement will be typeset if the manuscript is accepted for publication.<br><br>Before publication, authors are required to make all data underlying their findings fully available, without restriction. Review our PLOS Data Policy page for detailed information on this policy. Instructions for writing your Data Availability statement can be accessed via the Instructions link below.	The datasets used and/or analyzed during the current study have been attached as supplementary files.

Powered by Editorial Manager® and ProduXion Manager® from Aries Systems Corporation

Manuscript
Click here to access/download;Manuscript;Manuscript_FGS.docx

Profiling the knowledge of university female students and expertise of health care professionals in the management of female genital schistosomiasis in Anambra, South Eastern Nigeria.

Ogechukwu B. Aribodor[1]., Hammed O. Mogaji[2], Olabanji A. Surakat[3], Nwadiuto O. Azugo[1], Eunice C. Jacob[1], Emmanuel M. Obikwelu[4,] Obiageli Nebe[5] and Julie Jacobson[6]

1 Department of Zoology, Nnamdi Azikiwe University, Awka, Nigeria; **2** Parasitology and Epidemiology Unit, Department of Animal and Environmental Biology, Federal University Oye-Ekiti, Nigeria; **3** Department of Zoology, Department of Zoology, Faculty of Basic and Applied Sciences, Osun State University, Osogbo, Nigeria; **4** Neglected Tropical Diseases Unit, Anambra State Ministry of Health, Awka, Nigeria; **5** Neglected Tropical Diseases Unit, Federal Ministry of Health, Nigeria; **6** Bridges to Development, Vashon, Washington

Corresponding author: Ogechukwu Benedicta Aribodor; e-mail address: og.aribodor@unizik.edu.ng

Abstract

Background

Female genital schistosomiasis (FGS) is a largely neglected tropical disease, with little or no attention in the primary health care unit. Toward building momentum to address this challenge, we investigated the perception of university female students about FGS, as well as the expertise of health care professionals in Anambra State, Nigeria.

Methodology

We conducted a cross-sectional survey among 587 university female students and 65 health care professionals (HCPs) saddled with the responsibility of delivering care for schistosomiasis-affected persons. Pretested questionnaires were administered to document the awareness and knowledge about the disease. In addition, the expertise of HCPs vis-à-vis suspicion of FGS and management of FGS patients during routine health care service were documented. Data were analyzed in SPSS 20.0, and the confidence interval was set at 95%.

Results

Over half of the students recruited; 54.2% for schistosomiasis and 58.1% for FGS, were not aware of the disease. However, for HCPs, contrastingly high awareness about schistosomiasis (96.9%), but low awareness about FGS (61.9%) was observed. Knowledge about the transmission route for the disease was significantly poor among HCPs. A considerable proportion (>40%) of the HCPs never suspected schistosomiasis during routine clinical diagnosis of patients who presented probable FGS symptoms ($p < 0.05$). Similarly, only 28% were certain about the use of praziquantel for treating FGS cases, and about 35%-39% were uncertain of the eligibility criteria and dosage regimens. Commodities for managing FGS were also largely unavailable in about 39% of the health facilities where the HCPs work.

Conclusion

Our study has highlighted the significant lack of awareness and knowledge about FGS among university female students and HCPs in Anambra, Nigeria, and the resultant effect on under/misdiagnosis, and lack of treatment for FGS patients. It is therefore important to develop and incorporate FGS training materials in curricula of medical and para-medical university students, and training programs for HCPs during routine schistosomiasis control programs which has a wider coverage of engaged local health workforce.

Word count: 323

Authors' summary

We investigated the awareness and knowledge of schistosomiasis and Female Genital Schistosomiasis (FGS) among female students and Health Care Providers (HCPs) in Anambra State, Nigeria. Our findings shows that majority of the student participants have little knowledge about the diseases. However, the HCPs had knowledge of schistosomiasis, but knew very little about FGS. The HCPs also lack the capacity and resources to diagnose and manage FGS. This significant gap in knowledge, capacity and resources calls for development of FGS training materials, and its subsequent incorporation in curricula of medical/para-medical students, and training of HCPs during routine schistosomiasis control programs.

Background

Schistosomiasis is one of the most common neglected tropical diseases in the world, with over 206 million people affected, and about 2500 deaths in 2020 [1]. This disease is caused by parasitic water-borne trematodes of the genus Schistosoma, and over 90% of those affected reside in Africa. Two major Schistosoma species are common in this region; *S. mansoni* and *S. haematobium*, with the former and latter causing intestinal and urogenital schistosomiasis respectively [2]. The pathologies associated with both species vary depending on several factors, which are not limited to the severity of infection, migration of the worms through the organs and body tissues, and inflammatory responses to the presence of the eggs laid by the adult worms [3]. Intestinal schistosomiasis can result in symptoms such as abdominal pain, diarrhoea, blood in the stool, and in more severe cases, enlargement of the liver and spleen, a condition known as hepatosplenomegaly [3][4]. However, hematuria, which is classified as the passage of visible or invisible blood in urine is a common symptom of urogenital schistosomiasis [3]. Other complicated pathologies of urogenital schistosomiasis may include fibrosis of the bladder and ureter, kidney damage, and in more advanced cases cancer of the bladder [3]. Urogenital schistosomiasis may become more complex in females in a condition known as Female Genital Schistosomiasis (FGS).

FGS is a clinical condition used to describe the presence of trapped *S. haematobium* eggs, DNA, or characteristic clinical changes specifically in the genital tract of affected women, regardless of whether or not, the eggs are present in the urinary tract [5][6]. Many women acquire *S. haematobium* infection in childhood during domestic and recreational activities at infested water bodies, and about 75% of them may develop FGS [5][7]. Clinical manifestations such as vaginal bleeding, discharge, hematuria, dysuria, dyspareunia, and post-

coital bleeding are partly caused by trapped eggs which damage the genital mucosal linings with their characteristic terminal spine, and majorly the inflammatory responses to the embedded eggs [8]. This situation makes those affected more susceptible to HIV and Human Papillomavirus infections [9]. In addition, blockage of the uterus and/or fallopian tubes by the eggs or inflammatory responses to the egg may result in fertility problems. In adult females, eggs are commonly trapped in the cervix, vagina, and vulva, while vulvar lesions are common in younger females [5][6][10][11]. FGS lesions may present themselves as grainy sandy patches [6], homogenous yellow sandy patches [5], and rubbery papules.

As of 2020, there has been no assessment of FGS by the Global Burden of Disease study, either as a single entity or as part of the burden of schistosomiasis [12]. However, based on isolated reports, it is estimated that FGS affects about 56 million women and girls in Africa, and about 20 to 150 million females of all ages are estimated to be at risk of infection [13]. Interestingly, foreigners who visit infested freshwater bodies are also at risk [14]. In West Africa, Nigeria bears the highest burden of schistosomiasis, and invariably FGS. However, only two isolated studies in Ogun [15] and Anambra [16] have investigated the prevalence of FGS in Nigeria. These studies were conducted in rural areas with accommodating ecological conditions that tend to promote infections with schistosomiasis [15,16]. Interestingly, the preliminary findings from both states have spurred the interest of healthcare professionals and researchers to understand the epidemiology of FGS and also develop protocols aimed at highlighting other regions in the country where FGS is particularly endemic. This interest has been accompanied by investment from Bridges to Development through capacity building of health care professionals. Currently, FGS is largely neglected in the training curricula of medical and para-medical university students who subsequently transform into healthcare professionals. These gaps in training had contributed to misdiagnosis/classification of clinical

manifestations due to FGS, hence resulting in stigmatization, mental stress, and social exclusion of young females. This study, therefore, investigated the ~~perception of university students about FGS, as well as the expertise of health care professionals saddled with the responsibility of delivering health care services to those affected by FGS~~ in Anambra State, Nigeria. We believe our findings would be useful during consultation meetings as deliberations and strategies towards controlling FGS are mapped out in the state, and Nigeria at large.

Methodology

Ethics Statement and considerations

This study received ethical approval (COOUTH/CMAC/ETH.C/Vol.1/FN:04/0117) from the ethical review board of the Anambra State-owned Chukwuemeka Odumegwu Ojukwu University Teaching Hospital in Awka. Consent was sought from study participants who were prior to data collection. Written informed consent was obtained from study participants as applicable, and unique identifiers and a password-protected database were used to ensure anonymity and confidentiality through the study procedures.

Study area

Anambra is one of the 37 states in Nigeria, located in the southeastern part of the country, with 21 administrative regions known as local government areas (LGAs) (Fig 1). By population estimate, Anambra is ranked the 8th most populated state, with over 40% of its population living in rural areas where arable soil, tropical rain forest, topography, lakes, and river favors both farming/ fishing activities, as well as the transmission of schistosomiasis. Generally, the healthcare centers in rural areas lack trained healthcare personnel, are poorly

equipped, and with none or below standard laboratories which indicates an absence of public health outreaches and implementation of effective health outcomes.

Fig 1: Map of Nigeria showing Anambra State

Study design, mobilization, recruitment of study participants, and data collection

This study was cross-sectional in design, involving the use of quantitative methods to assess the perception and challenges of undergraduate students and health workers respectively. Following the guidelines of Bridges to Development and The Geneva Learning Foundation on the FGS Accelerated Scale Together (FAST) Package, an online questionnaire was developed during the First Scholar Workshop on Female Genital Schistosomiasis (FGS) and the First FGS Impact Accelerator meeting held between May and October 2021.

Upon completion and pilot testing of the questionnaire, the lockdown orders during the COVID-19 pandemic prevented physical interactions, as such, we administered the pretested electronic questionnaires to female students who are actively enrolled with the Nnamdi Azikiwe University in Awka. This prominent university has attracted residents living in rural areas across the LGAs in the State to migrate to Awka and other neighboring cities for educational and occupational-related activities. Females were invited to participate in the study through personal invitation letters sent to their institutional email, while WhatsApp broadcast messages, Facebook postings, and Telegram platforms were also utilized. An electronic packet containing a brief introductory note about the project, a question to seek consent, and a questionnaire were sent across the platforms. Only submissions of those who completed the consent questionnaire were included in the final analysis. Furthermore, health workers were interviewed physically during one of the routine stakeholder meetings for the

elimination of NTDs in Anambra State. Questionnaires were self-administered before the commencement of the stakeholders' meeting. Considering the literacy status of the participants, all electronic tools were designed and administered in the English language.

Data Management and Analysis

Data collected were imported into SPSS 20.0 statistical software (SPSS, USA) for analysis. Descriptive statistics such as frequencies and percentages were used to summarize and present the proportions of background and socio-demographic characteristics of the respondents. Chi-square tests were used to examine differences between variables of interest, with a significant level set at 95%.

Result

Demographic characteristics of the study participants

A total of 650; 587 university students and 65 healthcare professionals participated in this study. The majority of the female students (59%) were within the age category 20-24 years, followed by those between the age category 15 and 19 years. About 74% of them were from households that earn above the minimum national income of $50 per month. On the other hand, about 52% of the healthcare professionals recruited were females and 48% were males. About 56% of them were within the age category of 25 -45 years. Eight different fields of medical practice were represented, with a majority of the practitioners being medical doctors (36.9%), followed by laboratory scientists (21.5%), and community health workers (10.8%). Other specialization includes researcher, nurse, pharmacist, public health officer, and radiographer. About half of the practitioners have spent less than a decade in their current position, and about 87% of them work in a government-owned facility. However, only 46% of them have had experience with pelvic examination before (Table 1).

Table 1: Demographic characteristics of the study participants

	Frequency	%
**Female students (n=587)**		
**Age category (in years)**		
15-19	182	31
20-24	345	58.8
25-29	56	9.5
>30	4	0.7
**Average household income**		
<50$	148	25.2
>50$	433	73.8
Undefined	6	1.0
**Healthcare professionals (n=65)**		
**Gender**		
Male	31	47.7
Female	34	52.3
**Age category (in years)**		
20-24	7	10.8
25-29	14	21.5
30-34	7	10.8
35-39	7	10.8
40-44	11	16.9
45-49	7	10.8
>50	12	18.5
**Years of practice**		
<10	34	52.3
11-20	15	23.1
21-30	10	15.4
31-40	1	1.5
41-50	1	1.5
No response	4	6.2
**Specialization**		

Researcher [Academic]	3	4.6
Researcher [Clinical]	2	3.1
Community Health Worker	7	10.8
Laboratory Scientist	14	21.5
Medical Doctor	24	36.9
Nurse	5	7.6
Pharmacist	4	6.2
Public health officer	5	7.7
Radiographer	1	1.5
**Type of establishment**		
Government-owned	57	87.7
Private	8	12.3
**Conduct pelvic examinations?**		
**Yes**	30	46.2
**No**	35	53.8

Awareness about schistosomiasis and FGS among female students.

Of the 587 students interviewed, about half of them 318 (54.2%) affirmed they have heard of schistosomiasis. A majority reported their source of information was from the school environment 208 (65.6%), followed by social media 69 (21.8%), friends and family 26(8.2%), and through schistosomiasis, MDA campaigns 13 (4.1%). (Fig 2). However, when asked about FGS, only 246 (41.9%) of the participants have heard about the clinical condition. Among those that have heard, their source of information was from the school environment 132 (53.7%), followed by social media 66 (26.8%), friend and family 27 (11%) and through female genital schistosomiasis campaigns 17 (6.9%). (Fig 2).

Figure 2: *Awareness of schistosomiasis and FGS among university students*

Knowledge of health care professionals about schistosomiasis and FGS.

Of the 65 practitioners interviewed, 63 (96.9%) of them affirmed they have heard of schistosomiasis. The majority of these respondents 40 (63.5%) explained that it is a water-borne disease, followed by 17 (26.9%), who claimed it is transmitted by vectors. Other respondents claimed it can be transmitted through the feacal-oral pathway, 7 (11.1%) and person-to-person contact, (1, 1.5%). Only one respondent reported that it is caused by cercariae penetrating the skin of a susceptible host (Fig 3a). However, when asked about FGS, only 40 (61.9%) of the participants know about the disease. The majority of these respondents 32 (80%) explained that it is a water-borne disease, followed by sexual intercourse (13, 32.5%) and 11 (27.5%), who claimed it is a disease transmitted by vectors. Other respondents claimed it can be transmitted through the feacal-oral pathway, 4 (10%) and person-to-person contact, (1, 2.5%). Only one respondent reported that it is caused by the cercariae penetrating the skin of a susceptible host which later gets to the genitals (Fig 3).

Figure 3: Knowledge of health care professionals about schistosomiasis and FGS.

Prevalence of schistosomiasis suspicion among practitioners during routine practice.

Of the 65 practitioners interviewed, 41 (63.1%) of them provide patient-based care to people with venereal diseases, significant lower abdominal pain, and hematuria, respectively. However, 33 (50.8%) of them have provided care to patients presenting dysuria. The majority of the practitioners reported suspecting schistosomiasis infection during their diagnosis, with the majority of the suspicion attributed to patients who present with dysuria (60.6%), hematuria (58.5%), significant lower abdominal pain (56.1%) and venereal diseases (53.7%). Almost 90% of the practitioners who provide care for patients with venereal diseases have used praziquantel as a drug of choice when they suspect schistosomiasis. There were

significant differences in the proportions reported for schistosomiasis suspicion across the different types of diseases treated ($p < 0.05$) (Table 2).

Table 2: Prevalence of schistosomiasis suspicion among practitioners during routine practice.

		**Suspect a possible schistosomiasis infection in your diagnosis**			p-value
**Practitioners who treat; (N=65)**	NT	Yes	No	NA	
Venereal diseases	41(63.1)	22(53.7)	18(43.9)	1(2.4)	0.004
Significant lower abdominal pain	41(63.1)	23(56.1)	17(41.5)	1(2.4)	0.002
Hematuria	41(63.1)	24(58.5)	16(39.1)	1(2.4)	0.007
Dysuria	33(50.8)	20(60.6)	12(36.4)	1(3.0)	0.010
Use praziquantel to treat venereal diseases	24(36.9)	21(87.5)	3(12.5)	0(0)	0.000

N: Number of practitioners recruited; NT: Number of practitioners who treat one form of clinical condition; p-value is significant at 0.05

Management of FGS patients during routine health care service

About half of the practitioners were uncertain whether, or not they should quarantine patients with FGS. Similarly, only 28% of the practitioners affirmed that praziquantel is the only drug of choice for treating FGS (Table 3). A considerable proportion (39%) of them were uncertain if they were to treat pregnant women, and when to treat them during the pregnancy stage. In addition, about 35% of them do not know that the dosage of praziquantel varies across age categories (Table 3). Commodities for managing FGS were largely unavailable (39%) in the workplaces of the practitioners. However, about 29% reported having access to praziquantel in their workplace, while 20% reported having both praziquantel and schistosomiasis detection kits or urinalysis test strips and 11% reported having only test strips (Fig 4).

***Fig 4**: Availability of commodities at health care centers for FGS patients*

Table 3: Knowledge about the management of FGS patients during routine healthcare service

N=65	Frequency	%
**Persons affected with FGS should be quarantined.**		
Yes	1	1.5
No	37	56.9
Don’t know	20	30.8
Maybe	7	10.8
**Praziquantel is the only treatment for FGS.**		
Yes	18	27.7
No	22	33.8
I don’t know	25	38.5
**Praziquantel can be used to treat pregnant women.**		
Yes	40	61.5
No	25	38.5
**In which trimester can pregnant women take praziquantel?**		
First	2	3.1
Second	10	15.4
Third	22	33.8
All	18	27.7
None	13	20.0
**The praziquantel dosage is the same for adults and children**		
Yes	5	7.7
No	43	66.2
I don’t know	17	26.2

Participants' experiences during routine healthcare service on FGS-related symptoms

Of the eighty-three residents who presented a case of painful urination to the health care center, only 22 (26.5%) of them were asked if they had recent contact with fresh water bodies.

This is similar to 12 (38.7%) of the 31 residents who presented hematuria, 44 (23.5%) of 187 who presented lower abdominal pain, and 31 (37.3%) of the 83 residents who presented venereal diseases (Table 5).

Table 5: Participants' experiences during routine health care service on FGS-related symptoms

	**The practitioner asked about recent contact with freshwater**			
**Presented symptoms**	NE	Yes	No	p-value
Painful urination	83	22 (26.5)	61 (73.5)	0.458
Hematuria	31	12 (38.7)	19 (61.3)	0.035
Lower abdominal pain	187	44 (23.5)	143 (76.5)	0.786
Venereal diseases	83	31 (37.3)	52 (62.7)	0.000

p-value is significant at 0.05

Discussion

Schistosomiasis has been largely studied in Nigeria [17][18], and a network of partnerships between national and international agencies has invested commendable resources to control the menace of the disease [19][20]. In 2019, about 250 praziquantel tablets were administered in endemic communities, with the sole beneficiaries being children under age 15 [1]. Until recent, adults, most especially females of reproductive age (>15 years) have been largely neglected in schistosomiasis control programs [21], owing to the premise that mass administration of praziquantel to children under 15 is sufficient to reduce the burden of schistosomiasis in an endemic setting [22]. Unfortunately, women living in endemic regions could acquire *S. haematobium* infection in childhood [16][23][24], which may develop into female genital schistosomiasis (FGS) when left untreated [21][15][5][7]. The clinical manifestations and consequences of FGS, on both the sexual and reproductive health of

females, have been extensively described [8], and in recent times have gained public health traction [25].

However, despite the fact that FGS affects about 56 million women and girls in Africa, with about 20 to 150 million females estimated to be at risk, the disease remains largely neglected [13]. This is partly because the clinical manifestations are often misdiagnosed and confused with those of other sexually transmitted infections (STIs) [26], which in most cases do not only lead to undertreatment of genuine FGS cases but also stigmatization, mental stress, social exclusion and impaired life quality among young females who are wrongly misdiagnosed to have STIs [12][26]. Efforts targeted at addressing this capacity gap, most especially among healthcare professionals are emerging, for example with the FAST package implemented in some of Ghana and Madagascar [27] and other capacity-building workshops organized by Bridges to Development [28]. In this study, we, therefore, characterized the profiles of the university students vis-a-vis their knowledge and awareness about FGS, since they are most likely to take on healthcare-related jobs in the subsequent future. In addition, we assessed the expertise and challenges of healthcare professionals that are currently engaged in schistosomiasis control activities as a precursor to developing interventions to support the control of FGS.

Firstly, our findings revealed poor knowledge and awareness about schistosomiasis and FGS among university students, with a majority of those that have heard about the disease attributing the source of information to the school environment and social media. This reiterates the importance of incorporating FGS-related material into the medical training curricula of university students [25]. The potential of social media and radio programmes in promoting health-educational messages have been reported elsewhere [29][30] and can be

explored in this context to promote FGS. Similarly, we observed a contrastingly high awareness of schistosomiasis, but low awareness of FGS among healthcare professionals which is in line with previous reports [25][31]. A considerable proportion of our respondents attributed the cause of FGS to contact with water bodies and sexual intercourse, while only a few (2.5%) claimed it is a result of untreated *Schistosoma* infection. The misconception that FGS is transmitted via sexual intercourse needs to be addressed as it could have far-reaching implications on the socioeconomic life of affected women. It should therefore be advocated that FGS is a consequence of embedded *S. haematobium* eggs, DNA, or in the genital tract of affected women [5][6], and the condition is directly related to exposure to infected waterbodies harboring the *Bulinus* spp. More importantly, about 40% of the health care professionals claimed they had never suspected FGS among patients who reported venereal infections, lower abdominal pain, hematuria, and dysuria. Largely, 60-70% of the female residents also affirmed that healthcare professionals had never asked them about their history of contact with rivers when they presented symptoms such as painful urination, hematuria, lower abdominal pain, and venereal diseases to the clinic. The management of FGS was poor among the health care professionals, as a considerable proportion do not know the drug of choice for FGS, and the dosage requirement for treating adults, children, and pregnant women. This lack of awareness about FGS among healthcare workers has been reported elsewhere [25][31][15] and coupled with the lack of commodities including praziquantel tables, Kato Katz kits, urine filtration kits, and materials for colposcopy are the reasons why FGS remains a misunderstood and neglected disease. It is therefore important to complement discussion around increasing awareness and knowledge of health care professionals, with considerable investments and provision of adequate commodities for detecting and treating schistosomiasis and FGS.

Summarily, the lack of knowledge and expertise, among health care providers to appropriately diagnose FGS has resulted in under or misdiagnosis of the clinical conditions, hence, limiting access to treatment for women and girls suffering from this preventable and treatable disease. It is therefore important to consider interventions targeted at improving healthcare workers' knowledge by incorporating FGS-related material into medical training curricula [25] or more routinely into ongoing schistosomiasis control programme which are implemented at sub-district levels in endemic countries. The latter approach would allow wider coverage to the already engaged local health workforce within implementation units where the disease is most endemic. As previously highlighted by Jacobson, [25], this step would require identifying a clear and standardized set of learning outcomes; specifically, the competencies or behaviors required to adequately prevent, diagnose, and manage an FGS case. It is, therefore, significant to highlight that currently developed tools can be adapted for local pilot among healthcare professionals in the study area, to improve health workforce capacity and contribute to alleviating the neglect of FGS.

Conclusion

Our study has highlighted the significant lack of awareness and knowledge about FGS among university students and healthcare professionals in one of the southeastern states of Nigeria where the FGS burden has been previously mapped. In addition, we have highlighted the resultant effect of these gaps on underdiagnosis, misdiagnosis, and/or lack of or inappropriate treatment for FGS contributing to poor reproductive health outcomes in endemic areas. It is therefore important to develop and incorporate FGS training materials in training curricula of university students and training programs of health professionals during routine ongoing schistosomiasis control programs which have a wider coverage of engaged local health workforce within implementation units where the disease is most endemic.

Limitations of the study

This study was conducted during the COVID-19 pandemic with considerable restrictions on movements, hence we employed an electronic mode of data collection for undergraduate students recruited from public and government-owned tertiary institutions. In addition, we assessed participants' knowledge about schistosomiasis and FGS using a binary outcome, hence we cannot ascertain if respondents know the true definitions and conditions of the diseases.

Acknowledgments

We are grateful to the participants for their support. Our profound gratitude goes to the Geneva Learning Foundation for their collaboration in this study.

Declarations

Consent for publication

Not applicable

Availability of data and materials

The datasets used and/or analyzed during the current study have been attached as supplementary files.

Competing interests

The authors declare that they have no competing interest

Funding

Not applicable

Authors' contribution

OBA conceptualized the study. OBA, NOA, and ECJ prepared the protocol, while HOM,

OAS, EMO, ON, and JJ improved the protocol. OBA, NOA, and ECJ participated in field surveys and data collection. OBA, HOM, and OAS performed all statistical analyses and also prepared the first draft of the manuscript. All authors contributed to the development of the final manuscript and approved its submission.

REFERENCES

1. World Health Organization, "Ending the neglect to attain the Sustainable Development Goals: a road map for neglected tropical diseases 2021–2030," Geneva: World Health Organization, 2020. Licence: CC BY-NC-SA 3.0 IGO.
2. Colley DG, Bustinduy AL, Secor WE, King CH. Human schistosomiasis. *Lancet (London, England)*, 2014; 383(9936): 2253–2264. https://doi.org/10.1016/S0140-6736(13)61949-2.
3. World Health Organization, "Schistosomiasis," *World Health Organization*, 2021. [Online]. Available: https://www.who.int/news-room/fact-sheets/detail/schistosomiasis. Accessed 20 September 2021.
4. Jose RL. Acute schistosomiasis mansoni: revisited and reconsidered. Mem Inst Oswaldo Cruz, Rio de Janeiro, 2010; 105(4):422-435. doi.org/10.1590/S0074-02762010000400012.
5. Kjetland EF, Ndhlovu PD, Mduluza T, Gomo E, Gwanzura L, Mason PR, et al. Simple clinical manifestations of genital Schistosoma haematobium infection in rural Zimbabwean women. Am J Trop Med Hyg, 2005; 72(3): 311-319.
6. Kjetland EF, Poggensee G, Helling-Giese G, Richter J, Sjaastad A, Chitsulo L, et al. Female genital schistosomiasis due to Schistosoma haematobium. Clinical and parasitological findings in women in rural Malawi. Acta Trop, 1996; 62(4): 239-255.
7. Leutscher P, Ravaoalimalala VE, Raharisolo C, Ramarokoto CE, Rasendramino M, Raobelison A, et al. Clinical findings in female genital schistosomiasis in Madagascar. Trop Med Int Health, 1998; 3(4): 327-332.
8. Sturt AS, Webb EL, Francis SC, Hayes RJ, Bustinduy AL. Beyond the barrier: Female Genital Schistosomiasis as a potential risk factor for HIV-1 acquisition. Acta Tropica, 2020; 209(105524): https://doi.org/10.1016/j.actatropica.2020.105524.
9. Hegertun IEA, Sulheim GKM, Kleppa E, Zulu SG, Gundersen SG, Taylor M, et al. *S. haematobium* as a Common Cause of Genital Morbidity in Girls: Cross-sectional Study of Children in South Africa. PLoS Neglected Tropical Diseases, 2013; 7(3): e2104. https://doi.org/10.1371/journal.pntd.0002104.
10. Helling-Giese G, Kjetland EF, Gundersen SG, Poggensee G, Richter J, Krantz I, Feldmeier H. Schistosomiasis in women: manifestations in the upper reproductive tract. Acta Trop, 1996; 62(4): 225-238.

11. Swai B, Poggensee G, Mtweve S, Krantz I. Female genital schistosomiasis as evidence of a neglected cause for reproductive ill-health: a retrospective histopathological study from Tanzania. BMC Infect Dis, 2006; 6: 134.

12. Engels D, Hotez PJ, Ducker C, Gyapong M, Bustinduy AL, Secor WE, et al. Integration of prevention and control measures for female genital schistosomiasis, HIV and cervical cancer. Bulletin for World Health Organisation, 2020; 98(9): 615–624. 10.2471/BLT.20.252270.

13. Kukula VA, MacPherson EE, Tsey IH, Stothard JR, Theobald S, Gyapong M. A major hurdle in the elimination of urogenital schistosomiasis revealed: Identifying key gaps in knowledge and understanding of female genital schistosomiasis within communities and local health workers. PLoS Negl Trop Dis, 2019; 13(3): e0007207. https://doi.org/10.1371/journal.pntd.0007207.

14. Aribodor, OB. "Sneglefeber: Overset sygdom plager millioner af kvinder," *Forskerzonen*, 2022. [Online]. Available: https://videnskab.dk/forskerzonen/krop-sundhed/sneglefeber-overset-sygdom-plager-millioner-af-kvinder

15. Ekpo U, Odeyemi O, Sam-Wobo S, Onunkwor O, Mogaji H, Oluwole A, Abdussalam, HO, Stothard JR. Female genital schistosomiasis (FGS) in Ogun State, Nigeria: a pilot survey on genital symptoms and clinical findings. Parasitology Open, 2017; 3: E10: 1-9.

16. Aribodor OB, Okaka CE, Sam-Wobo SO, Okpala BC, Aribodor DN, Obikwelu E. Urinary Schistosomiasis and Primary Evidence of Female Genital Schistosomiasis among Pupils in Nsugbe Community, Anambra State, Nigeria. Nigerian Journal of Parasitology, 2021; 42(2): 294-402. 10.4314/njpar.v42i2.26.

17. Oyeyemi OT, Jeremias WJ, Grenfell RFQ. Schistosomiasis in Nigeria: Gleaning from the past to improve current efforts towards control. One Health, 2020; 11:100183. doi: 10.1016/j.onehlt.2020.100183.

18. Ezeh CO, Onyekwelu KC, Akinwale OP, Shan L, Wei H. Urinary schistosomiasis in Nigeria: a 50-year review of prevalence, distribution and disease burden. Parasite, 2019; 26:19. https://doi.org/10.1051/parasite/2019020

19. ESPEN, 2022. https://espen.afro.who.int/

20. Federal Ministry of Health (FMoH). Neglected Tropical Diseases Master Plan 2015–

2020. [Accessed: 15 January 2021]. Available at https://espen.afro.who.int/system/files/content/resources/NIGERIA_NTD_Master_Plan_2015_2020.pdf.

21. Mishra P, Colombe S, Paul N, Mlingi J, Tosiri I, Aristide C, et al. Insufficiency of annual praziquantel treatment to control Schistosoma mansoni infections in adult women: A longitudinal cohort study in rural Tanzania. PLoS Negl Trop Dis, 2019; 13(11): e0007844, https://doi.org/10.1371/journal.pntd.0007844
22. World Health Organization, "Helminth control in school-age children: a guide for managers of control programmes," 2011. [Online]. Available: https://www.who.int/neglected_diseases/resources/9789241548267/en/ [Accessed 15 April 2021].
23. Verani JR, Abudho B, Montgomery SP, Mwinzi PN, Shane HL, Butler SE, et al. Schistosomiasis among young children in Usoma, Kenya. Ame J of Trop Med Hyg, 2011; 84(5):787–791. https://doi.org/10.4269/ajtmh.2011.10-0685
24. Ekpo UF, Laja-Deile A, Oluwole AS, et al. Urinary schistosomiasis among preschool children in a rural community near Abeokuta. Nigeria. Parasites Vectors, 2010; 3, 58. https://doi.org/10.1186/1756-3305-3-58
25. Jacobson J, Pantelias A, Williamson M, Kjetland EF, Krentel A, Gyapong M, et al. Addressing a silent and neglected scourge in sexual and reproductive health in Sub-Saharan Africa by development of training competencies to improve prevention, diagnosis, and treatment of female genital schistosomiasis (FGS) for health workers. Reprod Health, 2022; 19:20. https://doi.org/10.1186/s12978-021-01252-2
26. Hotez PJ, Harrison W, Fenwick A, Bustinduy AL, Ducker C, Mbabazi PS, et al. Female genital schistosomiasis and HIV/AIDS: Reversing the neglect of girls and women. PLoS Negl Trop Dis, 13(4):e0007025.
27. "FAST Package - Dedicated to Combatting FGS," 2022. [Online]. Available: https://fastpackage.org/. [Accessed 23 June 2022].
28. Bridges to Development, "Advancing resilient communities on a path towards sustainable development," Bridges to Development, 2022. [Online]. Available: https://bridgestodevelopment.org. [Accessed 23 June 2021]
29. Mogaji HO, Odoh IM, Iyeh CI, Adeniran AA, Oyedeji SI, Okoh HI, et al. Attendee's awareness about preventive chemotherapy neglected tropical diseases (PC-NTD) control during the first world neglected tropical diseases day in Ekiti State, Nigeria.

PLoS Negl Trop Dis, 2021; 15(3): e0009315. https://doi.org/10.1371/journal.pntd.0009315
30. Advocacy partnership. TB/MDR-TB Advocacy Tool Kit. [Online]. 2012. Available at http://www.stoptb.org/assets/documents/global/awards/cfcs/tb_mdr%20advocacy%20tool%20kit.pdf.
31. Bustinduy AL, Randriansolo B, Sturt AS, Kayuni SA, Leutscher PDC, Webster BL, et al. An update on female and male genital schistosomiasis and a call to integrate efforts to escalate diagnosis, treatment and awareness in endemic and non-endemic settings: The time is now. Advances in Parasitology, 2022; 115:1-44. https://doi.org/10.1016/bs.apar.2021.12.003.

 Click here to access/download;Figure;Fig 1.docx

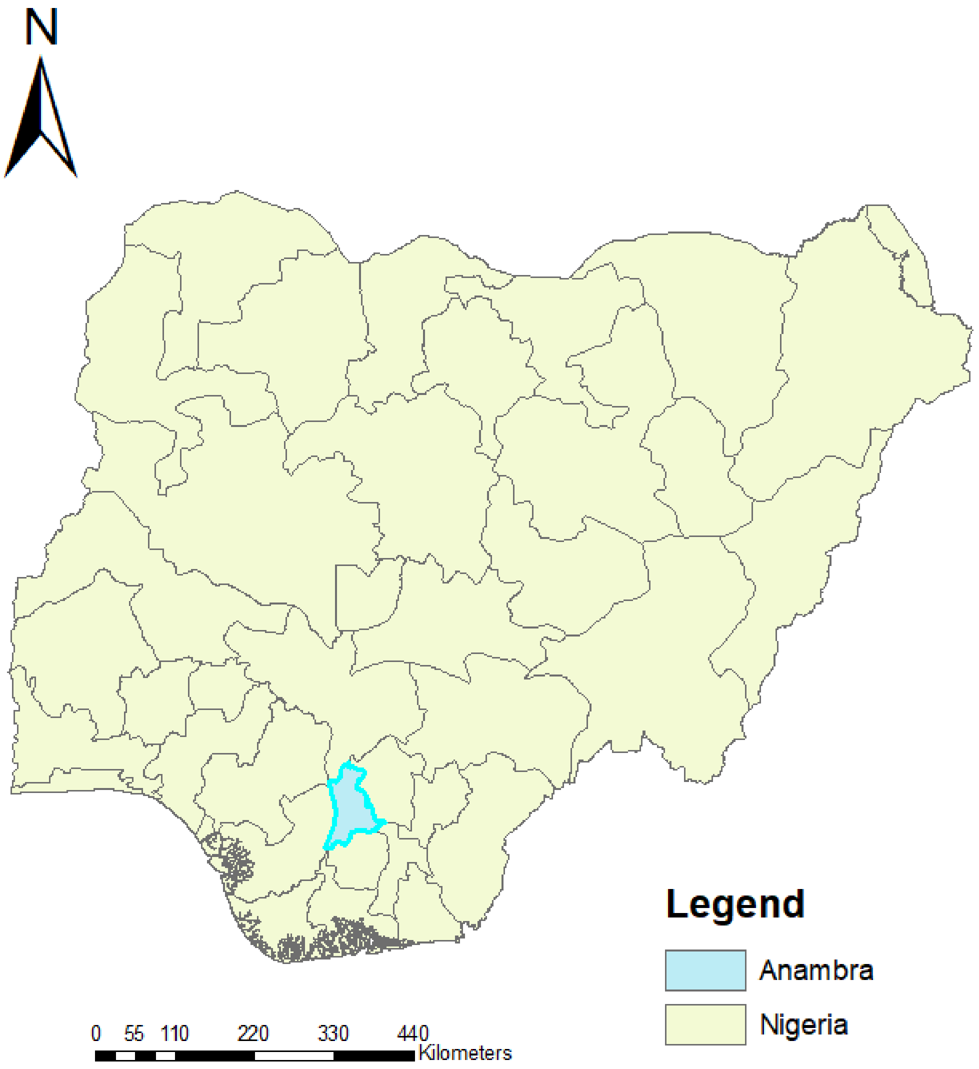

Figure 1: Map of Nigeria showing Anambra State

 Click here to access/download;Figure;Fig 2.docx

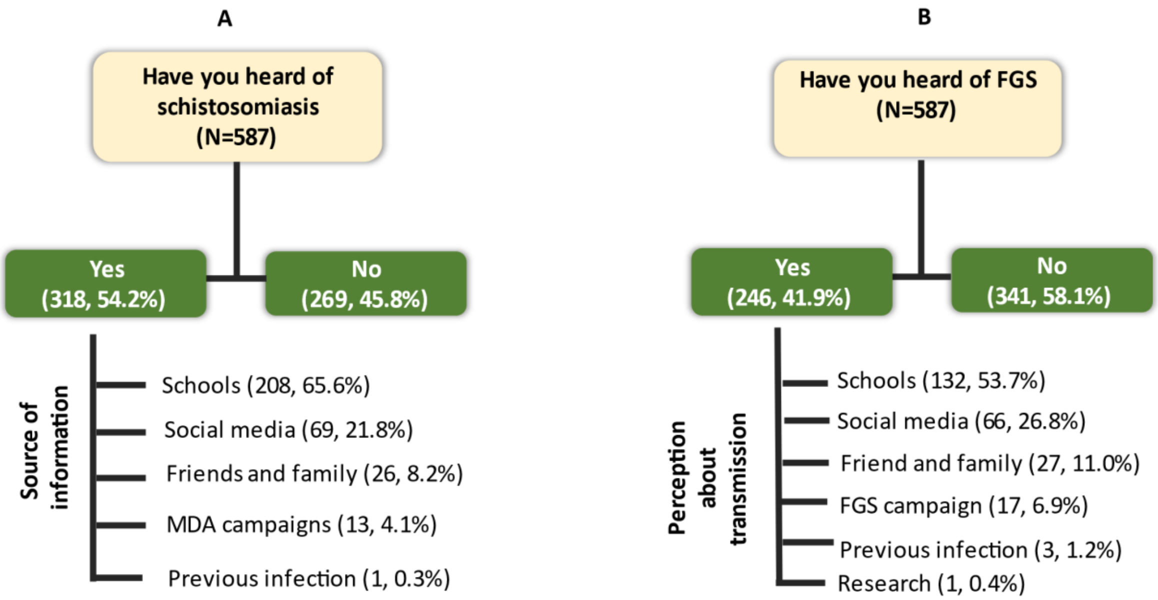

Figure 2: Awareness of schistosomiasis and FGS among university students

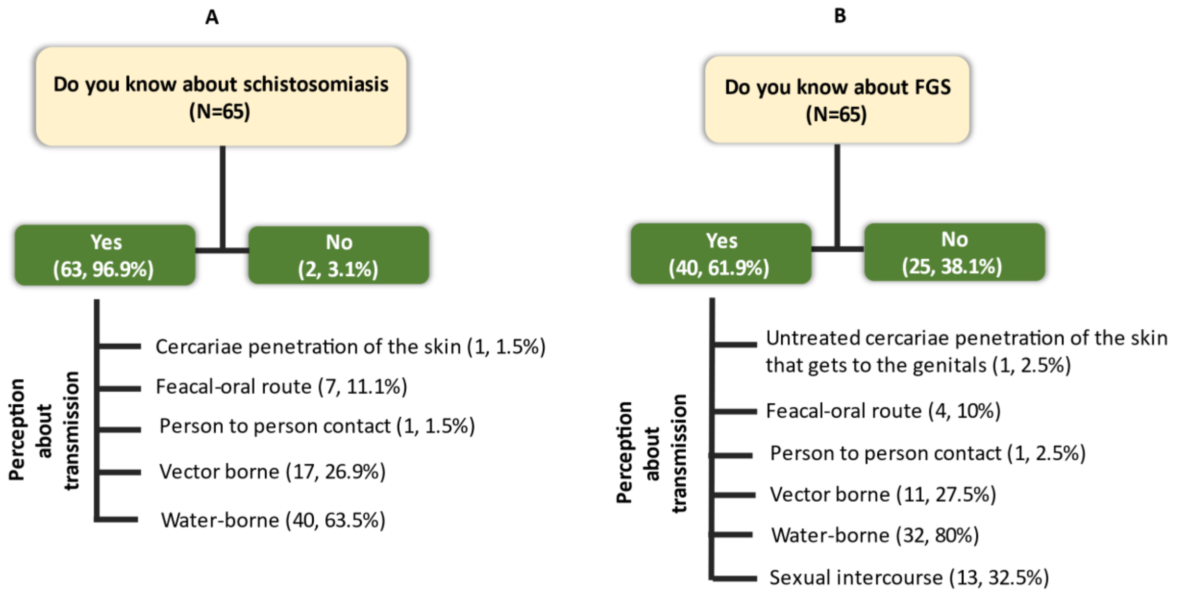

Figure 3: Knowledge of health care professionals about schistosomiasis and FGS.

 Click here to access/download;Figure;Fig 4.docx

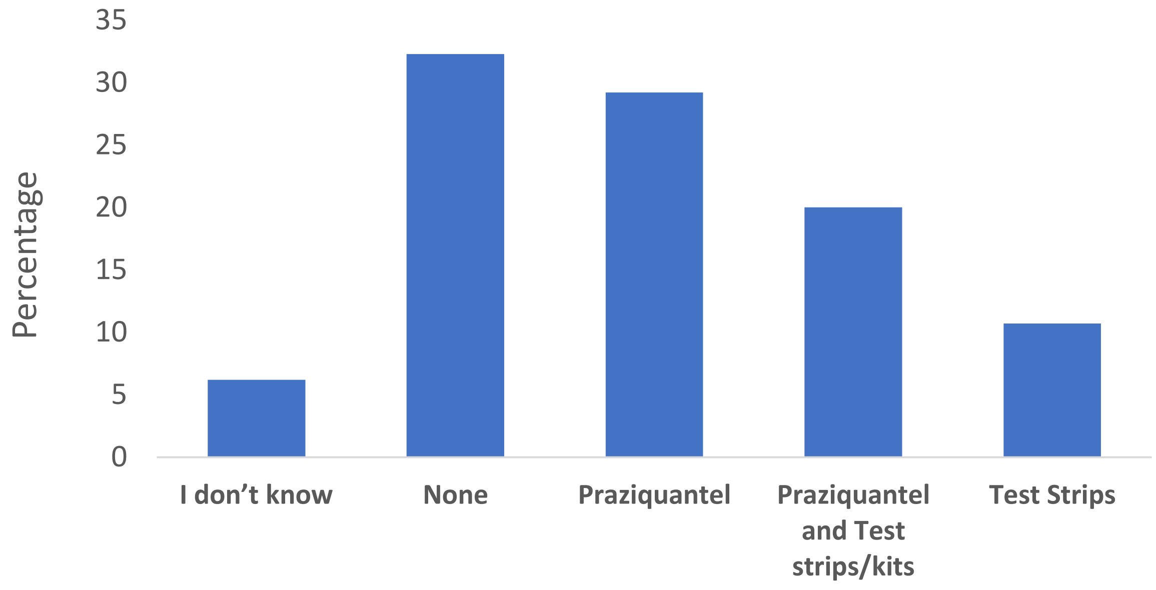

Figure 4: Availability of commodities at health care centers for FGS patients

Click here to access/download
Supporting Information
S1 FGS Schistomiasis Raw Data.xlsx

Click here to access/download
Supporting Information
S2 Healthworkers (Responses).xlsx

Click here to access/download
Supporting Information
S3 ETHICAL APPROVAL .jpg

Click here to access/download
Supporting Information
S3 APPROVAL 2.pdf
