# Supplementary material for: Profiling the knowledge of female medical/para-medical students, and expertise of health care professionals on female genital schistosomiasis in Anambra, South Eastern Nigeria
Source: PLoS Negl Trop Dis. 2023 Feb 16;17(2):e0011132. doi: 10.1371/journal.pntd.0011132 (PMC9977039; doi:10.1371/journal.pntd.0011132)
Supplement: Attachment — Submitted filename: Reviewer Comments.docx [file pntd.0011132.s002.docx]

This is an interesting topic, on the level of awareness and practices around schistosomiasis and female genital schistosomiasis among young female students and health practitioners in the South Eastern Nigeria. The authors have made a commendable attempt, but much still needs to be made clear, or presented differently, for a better understanding of their efforts. There is firstly an identified concern on the criteria and sampling strategy used, and how they arrived at the number of participants selected for interview; where this manuscript could benefit from a better presentation. Furthermore, the presentation of the results, and the translation of results to discussion, could benefit from more organization: here, the variables used and presented within the results such as gender, different age groups, years of practice and specialization of health practitioners questioned, could be considered further in analysis, to exploit better these results. There is need for a stronger discussion linked to the results presented here.

This manuscript will benefit from a major review.

--Abstract and through-out the manuscript.

It is understood from the concluding paragraph of the abstract (line 53), that the authors in referring to “female university students” may mean “medical and para-medical university students”. If this is the case, this manuscript may benefit in its over-all objective and precision if this is described as such early-on within the abstract and manuscript body.

--Line 118-124 and through-out the manuscript

Will benefit from references to support these statements

--Line 124-125

Once again please, are the authors referring to medical and para-medical students as supported on line 120-122?

--Line 127-129

How will this be useful? Please can the objective here be made clearer?

--Line 136-137

A word is lacking in the sentence.

--Line 137-138

‘As applicable’ is unclear. Does this mean informed consent was gotten ‘where applicable’? Thus, when was it not applicable?

--Line 142-149, and further within the manuscript

There is a need for adequate referencing/citing within the manuscript

--I53-175

Was a sample size calculated or how were participants selected, and the number ‘587’ and ‘65’ (line 34) arrived at? Sampling methods and reasoning applied here need to be defined and explained

--Line 153-154

Please the authors need to be precise on when this study was carried out

--Line 203,204, 207

What does ‘school environment’ refer to? Is this within curricula?

--Line 246-247

Question categories and reasoning behind this needs to be presented within the manuscript for clarity. For example, was ‘quarantine patients’ a category within the questioning, or was this gotten from respondents own understanding of the disease management? If it was a category, why?

--Line 249-251

Is this related to pediatric praziquantel? Or did researchers question respondents on praziquantel administration based on age? and not height/body weight? This is a bit confusing and might need to be clarified further.

--Line 251

‘commodities’ here refer to medical equipment; medication?

--Line 320-323

‘Female residents’ mentioned here. Are these the inhabitants of the study site community or study participants other than the female university students and health workers? The authors will need to present these respondents within the participant selection and description paragraph, before results (seen on line 268 and elsewhere within the results section), before discussing them here. Participant categories have been declared early on within the manuscript as female students and health workers, with their Knowledge, Attitudes, and Practices around schistosomiasis and female genital schistosomiasis questioned. There is need to present other ‘female residents’ and the questioning on their experiences when presenting at health centers or hospitals when showing schistosomiasis/FGS symptoms within the methods part of the manuscript as well.

Alternatively, if the description ‘female residents’ refers to ‘university students’ this has to be better written and consistent everywhere, to avoid misunderstanding, as well as the questioning on their hospital experiences brought out early-on when referring to the questions within the questionnaires administered.
