# Supplementary material for: Profiling the knowledge of female medical/para-medical students, and expertise of health care professionals on female genital schistosomiasis in Anambra, South Eastern Nigeria
Source: PLoS Negl Trop Dis. 2023 Feb 16;17(2):e0011132. doi: 10.1371/journal.pntd.0011132 (PMC9977039; doi:10.1371/journal.pntd.0011132)
Supplement: Attachment — Submitted filename: review_FGS.docx [file pntd.0011132.s004.docx]

**Abstract**

**Methodology:** Please explain why only female students were interviewed., Why should only female students be aware of the disease

**Line 46:** Please specify percentages

**Line 48:** Please specify percentages

**Authors summary:** please provide a broader picture of disease, do no repeat the abstract

**Background**

**Line 80-83:** please explain better on which factors the pathology depends on.

**Line 84-85:** please explain further what the long-term consequences of Schistosoma mansoni can be, beyond hepatosplenomegaly

**Line 85** : remove however.

**Line 89:** please explain in detail the pathophysiology of FGS

**Line: 93:** to my knowledge the sole presence of Schistosoma DNA does not define FGS, without any compatible clinical symptoms.

**Line 101**: please describe also pregnancy related problems.

**Line 104:** please provide Information on psychosocial consequences of FGS

**Line 119:** please explain what Bridges for development is

Line 122: this is an assumption. “Gaps in training might contribute” …

Line: 124: cite appropriate literature here

Line 125: please provide a rationale why only female students are of interest .

please provide information on how to diagnose FGS, questions in the methodology focus on the diagnosis of urinary schistosomiasis not FGS

**Methodology:**

Line: 136: remove who were

Line 138: please provide more details on data safety. Where did you safe electronic data?

**Results:**

Line 192: the group of health care professionals seems very heterogeneous, it seems unlikely to gain overview of knowledge from this group, why should a lab scientist be aware of treatment and diagnostic procedures on FGS. This is beyond the scope of his profession. Please exclude all profession which to not have patient contact and cannot potentially be confronted with patients having FGS. Or provide a specific reason to include them in your sample.

It would be interesting to see if there are knowledge difference between medical personnel and non medical personnel providing patient care

Line: 197: this is logical if you include professional groups which do not work in clinical care

Line 201: please stratify your results according to years of study in medicine. It might be that younger students know less simply because they did to touch up on the topic yet.

Line 239: table 2 show symptoms not diseases, Please specify if these cases you asked for FGS suspicion or schistosomiasis suspicion.

Line 252: please provide clear information that urinanalysis test strips and schisto detection kits do are commodities to provide diagnosis for urinary schisto but not for FGS. – This information needs to be placed in the background.

Line 251: Table 3 please provide this information only for personal having patient contact N 41

Line 266: Table 5 did these patient presented venereal disease or symptoms compatible with venereal disease which could be attributed to FGS.

Discussion:

Line 293: add Ref. Schuster et al <https://journals.plos.org/plosntds/article?id=10.1371/journal.pntd.0010901>

Line 295: wording

Line 299. This is wrong, some of the interviewed HCP are not engaged in schistosomiasis control activities.

Line 307: would it be possible to check of Schistosomiasis and FGS is part of the curriculum at the medical university? It might be useful to differentiate if it is taught but not thought well or if it is not taught. Please stratify your results by years of study. or if not possible by age. ( see below in methods section)

Line 319. Reported symptoms in line with veneral infection..

line 329: urinary filtration kits and kato katz does not allow to diagnose FGs but urinary or intestinal schistosomiasis. Urinary schistosomiasis is often associated with FGS. Solely the possibility to perform colposcopy together with the needed diagnostic competencies would allow to diagnose FGS. Therefore, it should be stressed that material and competences perform colposcopy as well the diagnostic knowledge to identify pathognomonic lesions on the cervix should be promoted.

Diagnostic helpers such as and diagnostic atlas with pathognomic images and Ai based application to support health workers in the diagnosis of FGS should be mentioned (<https://ieeexplore.ieee.org/document/9874491>)

**Limitations:**

Address the fact that only female students were interviewed
