# Supplementary material for: Profiling the knowledge of female medical/para-medical students, and expertise of health care professionals on female genital schistosomiasis in Anambra, South Eastern Nigeria
Source: PLoS Negl Trop Dis. 2023 Feb 16;17(2):e0011132. doi: 10.1371/journal.pntd.0011132 (PMC9977039; doi:10.1371/journal.pntd.0011132)
Supplement: Attachment — Submitted filename: Response to reviewers.docx [file pntd.0011132.s005.docx]

**RESPONSE TO REVIEWERS’ COMMENTS**

**REVIEWER 1:**

**General comment:** This is an interesting topic, on the level of awareness and practices around schistosomiasis and female genital schistosomiasis among young female students and health practitioners in the South Eastern Nigeria. The authors have made a commendable attempt, but much still needs to be made clear, or presented differently, for a better understanding of their efforts. There is firstly an identified concern on the criteria and sampling strategy used, and how they arrived at the number of participants selected for interview; where this manuscript could benefit from a better presentation. Furthermore, the presentation of the results, and the translation of results to discussion, could benefit from more organization: here, the variables used and presented within the results such as gender, different age groups, years of practice and specialization of health practitioners questioned, could be considered further in analysis, to exploit better these results. There is need
for a stronger discussion linked to the results presented here. This manuscript will benefit from a major review.

**Response 1**: We thank the reviewer for the great deal of effort expended in reviewing this manuscript and pointing out very critical points and areas that needed attention.

We have thus revised the manuscript accordingly, most precisely included new sub-sections, expanded our lines, revised sentences that were not clear enough and included several references as appropriate. Our point-by-point revisions can be found below in response to the individual comments raised by the reviewer

On the last comments of the reviewer about the need to perform additional analysis with the HCPs data. We have thus performed a regression analysis to model the relationship between year of study (for students), year of practice and expertise (for HCPs) on the knowledge about FGS and Schistosomiasis [as separate outcome variables]. We found that students in the upper years of study had very higher odds of been more informed for schistosomiasis, but we didn’t find any association with FGS, and the HCPs year of practice and expertise was also not associated with knowledge of either disease. We believe the very small sample size of HCPs recruited (n=65), might have influenced the outcome of this analysis. However, we thought (in future studies) of expanding the sampling frame to other healthcare professionals at regional level rather than the state level as we have done in this present study). We really appreciate the reviewer for pointing us towards the direction of an additional analysis.

**Comment 1:** No, the objectives are not very clear and no clear testable hypothesis is stated. There is need for a clearer objective, and hypothesis stated

**Response 1:** Thank you very much for your comment. We have revised the objective and added the target audience who most likely would immediately benefit from the results of the study

**Please see line 141-148**

We therefore aim to understand the knowledge and perception of FGS among medical and paramedical students who subsequently transform into HCPs, as well as expertise of current HCPs who are saddled with the responsibility of delivering care to FGS patients. These findings would be useful to support the call for local investments, collaborations and innovations, when deliberations towards controlling FGS are been mapped out by schistosomiasis control programmers, researchers and policy makers in the state, and Nigeria at large.

**Comment 2:** Yes, the study design is appropriate to address the stated objectives

**Response 2: Thank you for your comments**

**Comment 3:** No, the population is not clearly described at this time, and a bit confusing with sampling methods not described

**Response 3:** Thank you very much for your comment. We agree perfectly with you, and we have now revised the section entirely to show the details of our sampling approach. We appreciate the reviewer for this comment., which significantly added value to our manuscript.

Please see line 172-197

**Study design, mobilization, recruitment of study participants, and data collection**

This study was conceptualized following the guidelines of Bridges to Development, and The Geneva Learning Foundation on the FGS Accelerated Scale Together (FAST) Package. The study was designed as a cross-sectional survey to assess the perception and expertise of female MPMS and HCPs using quantitative methods. As such, two electronic questionnaires were developed during the First Scholar Workshop on Female Genital Schistosomiasis (FGS) and the First FGS Impact Accelerator meeting held between May and October 2021. Questionnaires were pilot tested among two external supervisors and 10 female students in Anambra, before deploying it for field use in October 2021.

However, the COVID-19 lockdown orders and closure of schools during the strike actions from academic unions prevented physical interactions. We therefore employed an electronic mode of enrollment and administration of questionnaires. We recruited medical and para-medical female students enrolled in Nnamdi Azikiwe University in Awka. This university is government owned, and with the lowest tuition fee in the state. As such, the university was purposively selected as the sampling site owing to her prominence in attracting residents living in all parts of the state, and country at large.

A minimum of 500 medical and paramedical female students from the faculty of biosciences, medical sciences and pharmacy were targeted for recruitment. These students were approached through the headship of faculty-based student unions, and subsequently invited to participate in the study through personal invitation letters sent to their institutional email. Follow-up recruitments were done through dedicated WhatsApp and Telegram platforms. An electronic packet that contains a brief introductory note about the project, a question to seek consent, and the main questionnaire were sent across the platforms. Only submissions of those who completed the consent questionnaire were included in the final analysis.

Furthermore, we targeted 50 of the 100 health care practitioners who operate at the State level, and are members of the schistosomiasis control committee. These healthcare practitioners (HCPs) were interviewed physically during one of the routine stakeholder meetings organized by the Anambra State Schistosomiasis control and elimination program. Questionnaires were self-administered to consenting stakeholders before the commencement of the meeting. Considering the literacy status of participants (MPMS and HCPs), all questionnaires were designed and administered in English language.

**Comment 4:** Statistical methods used are okay

**Response 4: Thank you for your comments**

**Comment 5;** No ethical concerns, but the presentation of the ethical statements could be made clearer

**Response 5:** Thank you very much for your comment. We agree perfectly with you, and we have now revised the section entirely. Please see line 151-157

**Ethics Statement and considerations**

This study received ethical approval (COOUTH/CMAC/ETH.C/Vol.1/FN:04/0117) from the ethical review board of Chukwuemeka Odumegwu Ojukwu University Teaching Hospital. Consent was sought from study participants prior to data collection, and only participants who provided written informed consent were recruited. Unique identifiers were used in place of participants’ name to ensure anonymity, while a password-protected database was used to archive all datasets to ensure confidentiality.

**Comment 6:** The analysis presented does not completely match the analysis plan, but can be reviewed. There is need for results to be reviewed and to represent study population description, and study objective questions as described in methods.

**Response 6:** Thank you very much for your comment. We agree perfectly with you, and we have now revised the section entirely to show our sampling details. (We explained this already in response 3 above). We have also added a new section on “study indicators” (line 187-204). We have also complemented the gaps in our result section by performing a regression analysis to model the relationship between year of study (for students), year of practice and expertise (for HCPs) on the knowledge about FGS and Schistosomiasis [as separate outcome variables]. Please see Table 2 and its description: Line 292 - 312

**Comment 7:** The results are not very well clearly and completely presented

**Response 7:** We have describe made necessary corrections (especially on the Table 5 about the female residents, which have been corrected as they are sub-set of female students). Also, we have explained above that we have made further analysis suggested by this reviewer and the 2 other reviewers.

**Comment 8:** Yes, figures and tables are descriptive, though the variables presented in tables are not analyzed in the discussion.

**Response 8:** Thank you for your comments. We have now added some significant lines (393-403, 423-430, 445-448) to provide more clarity to the discussion page Actually, we employed a deductive method of discussion for all the results in our manuscript. As such, rather than analyzing (table by table) i.e., repeating the result section in the discussion page, we summarized the most important results and discussed their implications in a singular flow, as it relates with the hypothesis set out.

**Comment 9:** The 'Author summary' could be reviewed to present more background of the identified problem and contextualized within the selected study site.

**Response 9:** Thank you very much for your comment. We have revised the author summary accordingly. Please see line 65-81

Female genital schistosomiasis (FGS) is a neglected disease that is often mistaken for other sexually transmitted infections. The disease has thus received little attention at the primary health care unit until recent. To support the emerging momentum been built towards controlling this disease, we conducted this study to understand the knowledge and perception of FGS among medical and paramedical students (MPMS) who subsequently transform into HCPs, as well as expertise of current HCPs who are saddled with the responsibility of delivering care to FGS patients. 587 MPMS and 65 HCPs were interviewed in Anambra State, using well designed questionnaires. Our findings show that majority of the MPMS were not aware of schistosomiasis and FGS, but students in the 6^th^ year of study have higher chances of been informed about schistosomiasis. However, almost all the HCPs were aware of schistosomiasis, but FGS awareness was very low. The HCPs also lack the capacity and resources to diagnose and manage FGS. This significant gap in knowledge, capacity and resources calls for development of FGS training materials, and its subsequent incorporation in curricula of medical students, and training of HCPs during routine schistosomiasis control programs. This should be complemented with the provision of necessary diagnostics to perform colposcopy, as well as building competence to diagnose pathognomonic lesions using diagnostic atlas or Artificial Intelligence (AI).

**Comment 10;** --Abstract and through-out the manuscript.
It is understood from the concluding paragraph of the abstract (line 53), that the authors in referring to “female university students” may mean “medical and para-medical university students”. If this is the case, this manuscript may benefit in its over-all objective and precision if this is described as such early-on within the abstract and manuscript body.

**Response 10:** Thank you very much for your comment. This is absolutely true, and we have corrected the entire manuscript by replacing “female university students” with “medical and para-medical university students”. We replaced this word with an acronym “MPMS” just as we did for health care professionals “HCPs”

**Comment 11; --**Line 118-124 and through-out the manuscript. Will benefit from references to support these statements

**Response 11:** Thank you very much for your comment. We have included an additional reference on line 120, and also edited the sentence on line 122 to reflect our concerns that the gaps in training of medical and para-medical students may contribute to contribute to misdiagnosis/classification of clinical manifestations due to FGS

Please see the revisions below: 134-142

This interest has been accompanied by investment in capacity building of health care professionals from Bridges to Development, a nonprofit and tax-exempt organization in Switzerland and the United State of America, focused on advancing economic and social well-being of women and girls [17]. Currently, FGS is largely neglected in the training curricula of medical and para-medical university students (MPMS) who subsequently transform into health care professionals (HCPs). These training gaps might contribute to misdiagnosis/classification of clinical manifestations due to FGS, resulting in stigmatization, mental stress, and social exclusion of young females [11, 12, 13].

**Comment 12;** --Line 124-125
Once again please, are the authors referring to medical and para-medical students as supported on line 120-122?

**Response 12:** Thank you very much for your comment. We have improved our manuscript accordingly and replace the word female students with female medical and paramedical students

**Please see line 141-148**

We therefore aim to understand the knowledge and perception of FGS among medical and paramedical students who subsequently transform into HCPs, as well as expertise of current HCPs who are saddled with the responsibility of delivering care to FGS patients. These findings would be useful to support the call for local investments, collaborations and innovations, when deliberations towards controlling FGS are been mapped out by schistosomiasis control programmers, researchers and policy makers in the state, and Nigeria at large.

**Comment 13;** --Line 127-129. How will this be useful? Please can the objective here be made clearer?

**Response 13:** Thank you very much for your comment. We have added the target audience who most likely would immediately benefit from the results of the study. We have also revised the entire line to make our objective more clearer.

**Please see line 141-148**

We therefore aim to understand the knowledge and perception of FGS among medical and paramedical students who subsequently transform into HCPs, as well as expertise of current HCPs who are saddled with the responsibility of delivering care to FGS patients. These findings would be useful to support the call for local investments, collaborations and innovations, when deliberations towards controlling FGS are been mapped out by schistosomiasis control programmers, researchers and policy makers in the state, and Nigeria at large.

**Comment 14;** --Line 136-137. A word is lacking in the sentence.

**Response 14:** Thank you very much for your comment. We have revised the section to address the missing word: Please see line 152-158

This study received ethical approval (COOUTH/CMAC/ETH.C/Vol.1/FN:04/0117) from the ethical review board of Chukwuemeka Odumegwu Ojukwu University Teaching Hospital. Consent was sought from study participants prior to data collection, and only participants who provided written informed consent were recruited. Unique identifiers were used in place of participants’ name to ensure anonymity, while a password-protected database was used to archive all datasets to ensure confidentiality.

**Comment 15;** --Line 137-138. ‘As applicable’ is unclear. Does this mean informed consent was gotten ‘where applicable’? Thus, when was it not applicable?

**Response 15:** Thank you very much for your comment. We have revised the section to address the wrong use of “as applicable”: Please see line 152-158

This study received ethical approval (COOUTH/CMAC/ETH.C/Vol.1/FN:04/0117) from the ethical review board of Chukwuemeka Odumegwu Ojukwu University Teaching Hospital. Consent was sought from study participants prior to data collection, and only participants who provided written informed consent were recruited. Unique identifiers were used in place of participants’ name to ensure anonymity, while a password-protected database was used to archive all datasets to ensure confidentiality.

**Comment 16**--Line 142-149, and further within the manuscript. There is a need for adequate referencing/citing within the manuscript

**Response 16:** Thank you very much for the wonderful observation. We have restructured the entire paragraph and provided the necessary citations. We have added 6 new citations.

Please see line 160-169

Anambra is one of the 37 states in Nigeria, located in the southeastern part of the country, with 21 administrative regions known as local government areas (LGAs) (Fig 1). By population estimate, Anambra is ranked the 14^th^ most populated state [18], with a very low rurality index following Lagos [19]. However, the presence of arable soil, tropical rain forest, topography, lakes, and river favors both farming/ fishing activities, as well as the transmission of schistosomiasis [20,21]. In 2017, the most recent date in which statistics are available, there were over 590 public and 1600 private healthcare facilities operating in Anambra state [22], however, there are concerns that healthcare centers in rural areas lack trained healthcare personnel [23].

**Comment 17**--I53-175 Was a sample size calculated or how were participants selected, and the number ‘587’ and ‘65’ (line 34) arrived at? Sampling methods and reasoning applied here need to be defined and explained

**Response 17:** Thank you very much for your comment. We have now revised the section entirely to show the details of our sampling approach. We appreciate the reviewer for this comment. Our sampling size was a minimum of 500 students from the study location. However, because we targeted high-level health care practitioners, we were only limited within the sampling frame of practitioners who operate at the State level on NTDs (<100). We consider this limited size a limitation, and as a way forward in subsequent studies, we thought of expanding the sampling frame to other healthcare professionals at regional level rather than the state level as we have done in this present study.

Please see line 173-207

**Study design, mobilization, recruitment of study participants, and data collection**

This study was conceptualized following the guidelines of Bridges to Development, and The Geneva Learning Foundation on the FGS Accelerated Scale Together (FAST) Package. The study was designed as a cross-sectional survey to assess the perception and expertise of female MPMS and HCPs using quantitative methods. Two electronic questionnaires were developed during the First Scholar Workshop on Female Genital Schistosomiasis (FGS) (<https://www.learning.foundation/female-genital-schistosomiasis>) and the First FGS Impact Accelerator meeting (<https://www.learning.foundation/fgs>) held between May and October 2021. Questionnaires were pilot tested among two external supervisors and 10 female students in Anambra, before deploying it for field use in October 2021. However, physical interactions were impossible in October, because of COVID-19 lockdown orders and closure of schools during the strike actions by academic unions across all government -owned universities in the country. As such, we employed electronic mode of enrollment and administration of questionnaires. We recruited medical and para-medical female students enrolled in Nnamdi Azikiwe University in Awka. This university was purposively selected because it is government-owned, and with the largest enrollment due to its low tuition fee when compared to other universities in the state.

A minimum of 500 medical and paramedical female students from the three faculties; biosciences, medical sciences and pharmacy, were targeted for recruitment. These students were approached through the headship of faculty-based student unions, and subsequently invited to participate in the study through personal invitation letters sent to their institutional email. Follow-up recruitments were done through dedicated WhatsApp and Telegram platforms. An electronic packet that contains a brief introductory note about the project, a question to seek consent, and the main questionnaire were sent across the platforms. Only submissions of those who completed the consent questionnaire were included in the final analysis.

Furthermore, we targeted 50 of the 100 health care practitioners who operate at the State level, and are members of the schistosomiasis control committee. These healthcare practitioners (HCPs) were interviewed physically during one of the routine stakeholder meetings organized by the Anambra State Schistosomiasis control and elimination program. Questionnaires were self-administered to consenting stakeholders before the commencement of the meeting. Considering the literacy status of participants (MPMS and HCPs), all questionnaires were designed and administered in English language.

**Comment 18**--Line 153-154
Please the authors need to be precise on when this study was carried out

**Response 18:** Thank you very much for your comment. We have now revised the section to show the details of when the study was conducted.

Please see below Line 181-183

Questionnaires were pilot tested among two external supervisors and a 10 of female students in Anambra, before deploying it for field use in October 2021.

**Comment 19**--Line 203,204, 207
What does ‘school environment’ refer to? Is this within curricula?

**Response 19:** Yes, because we recruited medical and para-medical students, some of them learnt about schistosomiasis from within the school. As far as we know the Nigerian curricula cover schistosomiasis in basic biology courses in the first year, and subsequently in an advanced parasitology course, however, our questionnaire did not probe further on what (exactly) was the source of knowledge, as we understand their responses may be subject to recall bias. We have included this explanation in the discussion lines 391-401

**Comment 20**--Line 246-247
Question categories and reasoning behind this needs to be presented within the manuscript for clarity. For example, was ‘quarantine patients’ a category within the questioning, or was this gotten from respondents own understanding of the disease management? If it was a category, why?

**Response 20:** Thank you very much for your comments. We agree with you, and we have added a new sub-section “Study indicators”. Here we explained in details the indicators used, and also added a supplementary file to the tools. Because of the recent pandemic, and the undeniable fact that “quarantine” has become a common a word and practice in most parts of the world, we thought of including a question around this on the tool for the practitioners to see if they perceive FGS as a serious threat to other persons, and also see if they know the right treatment options (which of course is not to quarantine the patients). As such, we added a question category on quarantine, and expected a negative response. However, we were surprised to see about 41% of the practitioners saying they “don’t know if FGS patients should be quarantined”. This further strengthens our resolve on building capacity of medical practitioners on FGS.

Please see line 210-226 for the study indicators

**Study indicators**

We used two separate tools (See S1 File) and (See S2 File) to document responses from female MPMS and HCPs, respectively. For HCPs, demographic information including age, gender, profession, year of practice and place of work were documented. In addition, we collected information on HCPs expertise on a range of symptoms/condition that overlaps with FGS pathology. These included; (i) if the practitioner performs pelvic examination, (ii) treat venereal diseases, (ii) lower abdominal pain, (iv) dysuria or (v) hematuria. Knowledge of HCPs about schistosomiasis and FGS were also documented, with emphasis on (i) awareness, (ii) source of information, (iii) transmission routes, (iv) preventive practices, (v) treatment choices with emphasis on pregnant women, and (vi) dosage requirements for children and adult. HCPs were also asked if FGS patients should be quarantined and if care commodities for FGS patients are available in their place of work (See S1 File)

Similarly for female MPMS, we collected information on age, religion, parents’ occupation and income. In addition, knowledge about schistosomiasis and FGS were documented, with emphasis on awareness and source of information. We also documented if MPMS (i) have ever been treated with praziquantel, (ii) had contact with freshwater, or (iii) had experienced any of the following symptoms that overlaps with FGS pathology; hematuria, painful urination, lower abdominal pain and venereal diseases (See S2 File).

**Comment 21**--Line 249-251
Is this related to pediatric praziquantel? Or did researchers question respondents on praziquantel administration based on age? and not height/body weight? This is a bit confusing and might need to be clarified further.

**Response 21:** Thank you for your comments. Praziquantel dosage is based on height/weight using the dose pole. As such, we asked “Is the dose for Praziquantel the same for a child as in an adult? and three level options were provided; Yes, No, and I don’t know. (with the correct option been “No’’). We have thus added the sub-section on “study indicators” to further guide readers and we have also re-structured the sentence to provide the needed clarifications.

Please see Line 335-336:

In addition, about 35% of them do not know the dosage requirements for praziquantel tablets (Table 3).

**Comment 22**--Line 251.‘commodities’ here refer to medical equipment; medication?

**Response 22:** Yes, thank you for this comment. We have improved the lines accordingly. Please see lines 337-339

Commodities such as medications and equipment for managing FGS were largely unavailable (39%) in the workplaces of the practitioners.

**Comment 23**--Line 320-323
‘Female residents’ mentioned here. Are these the inhabitants of the study site community or study participants other than the female university students and health workers? The authors will need to present these respondents within the participant selection and description paragraph, before results (seen on line 268 and elsewhere within the results section), before discussing them here. Participant categories have been declared early on within the manuscript as female students and health workers, with their Knowledge, Attitudes, and Practices around schistosomiasis and female genital schistosomiasis questioned. There is need to present other ‘female residents’ and the questioning on their experiences when presenting at health centers or hospitals when showing schistosomiasis/FGS symptoms within the methods part of the manuscript as well. Alternatively, if the description ‘female residents’ refers to ‘university students’ this has to be better written and consistent everywhere, to avoid misunderstanding, as well as the questioning on their hospital experiences brought out early-on when referring to the questions within the questionnaires administered.

**Response 22:** Thank you for this comment. We apologize for the mix-up, and absolutely agree with the reviewer. The respondents reported here on Line 268, are sub-set of the 587 female students in this study. From our analysis, a sub-set of the 587 students reported they had hematuria (n=31), painful urination (n=83), lower abdominal pain (n=187) and venereal diseases (n=83), and that they recently sought care from medical practitioners. As such, their data were separated for an in-depth analysis to document their experience with the practitioner who cared for them.

Please see line 352-358

Of the 587 female MPMS, 83 presented a case of painful urination to the health care center, and only 22 (26.5%) of them were asked if they had recent contact with fresh water bodies (p=0.458). Similarly, only 12 (38.7%) of the 31 students who presented hematuria were asked (p=0.035). In addition, only 44 (23.5%) of 187 who presented lower abdominal pain were asked (p=0.786), and only 31 (37.3%) of the 83 residents who presented symptoms of venereal diseases were asked (p=0.000) (Table 5).

Also, see line 412-415

Largely, 60-70% of the female students who reported painful urination, hematuria, lower abdominal pain, and venereal diseases also affirmed that healthcare professionals had never asked them about their history of contact with rivers when they sought medical care.

**REVIEWER 2:**

**General comment:** The manuscript should be accepted, after revision as recommended on the attached pdf version. It provides an insight into the understanding of the impact of the knowledge gap among health care professionals and the need to address this gap during undergraduate study. The study findings provide opportunities for NTD programmes to advocate for more resources and integration with STIs and reproductive health programmes. More research in this area is still needed to compliment these findings.

**Response:** We thank the reviewer for the great deal of effort expended in reviewing this manuscript and pointing out very critical points and areas that needed attention. Your contributions are well appreciated.

**Comment 1:** The objectives are clearly articulated, but the author needs to be more specific.

**Response 1:** Thank you very much for your comment. We have revised the objective to make it more specific, we also added the target audience who most likely would immediately benefit from the results of the study.

**Please see line 142-148**

We therefore aim to understand the knowledge and perception of FGS among medical and paramedical students who subsequently transform into HCPs, as well as expertise of current HCPs who are saddled with the responsibility of delivering care to FGS patients. These findings would be useful to support the call for local investments, collaborations and innovations, when deliberations towards controlling FGS are been mapped out by schistosomiasis control programmers, researchers and policy makers in the state, and Nigeria at large.

.

**Comment 2:** The study design is appropriate

**Response 2:** Thank you very much for your comment.

**Comment 3:** The population is clearly defined, need to be specific for the students enrolled

**Response 3:** Thank you very much for your comment. We agree perfectly with you, and we have now revised the methodology section entirely to show the details of our sampling approach and clearly define the population. We appreciate the reviewer for this comment., which significantly added value to our manuscript

Please see line 173-207

**Study design, mobilization, recruitment of study participants, and data collection**

This study was conceptualized following the guidelines of Bridges to Development, and The Geneva Learning Foundation on the FGS Accelerated Scale Together (FAST) Package. The study was designed as a cross-sectional survey to assess the perception and expertise of female MPMS and HCPs using quantitative methods. Two electronic questionnaires were developed during the First Scholar Workshop on Female Genital Schistosomiasis (FGS) (<https://www.learning.foundation/female-genital-schistosomiasis>) and the First FGS Impact Accelerator meeting (<https://www.learning.foundation/fgs>) held between May and October 2021. Questionnaires were pilot tested among two external supervisors and 10 female students in Anambra, before deploying it for field use in October 2021. However, physical interactions were impossible in October, because of COVID-19 lockdown orders and closure of schools during the strike actions by academic unions across all government -owned universities in the country. As such, we employed electronic mode of enrollment and administration of questionnaires. We recruited medical and para-medical female students enrolled in Nnamdi Azikiwe University in Awka. This university was purposively selected because it is government-owned, and with the largest enrollment due to its low tuition fee when compared to other universities in the state.

A minimum of 500 medical and paramedical female students from the three faculties; biosciences, medical sciences and pharmacy, were targeted for recruitment. These students were approached through the headship of faculty-based student unions, and subsequently invited to participate in the study through personal invitation letters sent to their institutional email. Follow-up recruitments were done through dedicated WhatsApp and Telegram platforms. An electronic packet that contains a brief introductory note about the project, a question to seek consent, and the main questionnaire were sent across the platforms. Only submissions of those who completed the consent questionnaire were included in the final analysis.

Furthermore, we targeted 50 of the 100 health care practitioners who operate at the State level, and are members of the schistosomiasis control committee. These healthcare practitioners (HCPs) were interviewed physically during one of the routine stakeholder meetings organized by the Anambra State Schistosomiasis control and elimination program. Questionnaires were self-administered to consenting stakeholders before the commencement of the meeting. Considering the literacy status of participants (MPMS and HCPs), all questionnaires were designed and administered in English language.

**Comment 4:** Sample size is sufficient

**Response 4:** Thank you very much for your comment.

**Comment 5**: Statistical analysis are correct

**Response 5:** Thank you very much for your comment.

**Comment 6:** Ethics procedures were followed

**Response 6:** Thank you very much for your comment.

**Comment 7:** The results match the analysis plan but the author needs to revise the text as recommended, in order to be clear. Table and figures are clear.

**Response 7:** Thank you very much for your comment. We have thus revised the manuscript accordingly, most precisely included new sub-sections, expanded our line, revised sentences that were not clear enough and included references as appropriate. Our point-by-point revisions can be found below in response to the individual comments raised by the reviewer

**Comment 8:** Conclusion is supported by data presented. Study limitations are clearly described. The authors discussed how the data can be helpful in advancing our understanding of the impact of FGS knowledge gap. The public relevance is addressed

**Response 8:** We thank the reviewer for the great deal of effort expended in reviewing this manuscript and pointing out very critical points and areas that needed attention. Your contributions are well appreciated.

**Comment 9:** Page 1:Abstract: Towards

**Response 9:** Thank you very much for your comment. We have revised the manuscript accordingly.

**Comment 10:** Page 1:Abstract Results: Over half of the students recruited were not aware of the disease (54.2% for schistosomiasis and 58.1% for FGS). However, a large proportion of HCPs (96.9%) showed high awarenes of schistosomiasis, while 61.9% of HCPs showed awareness of FGS.

How was high awareness measures? Some awareness are not measured.

**Response 10:** Thank you very much for your comment. We consider high awareness to be above 80%, but this is just based on authors convenience as there are no standard metrics.

**Comment 11:** Page 1: Abstract Results; If awareness was high, how come >40% HCPs never suspected the disease and only 28% were certain about the use of praziquantel

treatment?

**Response 11:** Thank you very much for your comment.

In this study, we asked just a simple question to assess the knowledge of the participants; if they know about FGS and Schistosomiasis, with 3 level options as responses (Yes, No, I don’t know). We have included in the limitation section that this metric of assessing knowledge is subject to bias, and we are more comfortable with using this question to judge the awareness of the respondents about the disease, but not their knowledge per say. This was evidently shown when we asked the respondents about the transmission routes and majority of them who affirmed to know about the diseases, gave wrong responses on the transmission route. Clearly, our findings support existing theories that awareness is not always related to knowledge, and hence supports the need to invest in building the capacity of HCPs.

**Comment 12:** Page 1:Abstract Conclusion: Is misdiagnosis related to lack of knowledge among students? Are these medical students who see patients at the health care facilities? If these are medical students, maybe it needs to be highlighted in the topic and under methodology.

**Response 12:** Thank you very much for this valuable comment. The students are female medical and paramedical students under training in the university. But a sub-set of them who had FGS related symptoms visited the health center to sought medical care, and majority of them reported the medical practitioner never asked them questions related to schistosomiasis during diagnosis. However, we have revised the conclusion entirely,

Please see below Lines 54-59

Awareness and knowledge about FGS among MPMS and HCPs were poor in Anambra, Nigeria. It is therefore important to invest in innovative methods of building capacity of MPMS and HCPs, with complementary provision of necessary diagnostics to perform colposcopy, as well as competence to diagnose pathognomonic lesions using diagnostic atlas or Artificial Intelligence (AI).

**Comment 13:** Page 4: Line 5 Remove the fullstop

**Response 13:** Thank you very much for your comment. We have revised the manuscript accordingly.

**Comment 14:** Page 4, Line 6: Put the comma at the right place

**Response 14:** Thank you very much for your comment. We have revised the manuscript accordingly.

**Comment 15:** Page 6, Line 65: Remove S

**Response 15:** Thank you very much for your comment. We have revised the manuscript accordingly.

**Comment 16:** Page 6 Line 66: How did you measure the knowledge (little knowledge and had knowledge)?

**Response 16:** Thank you very much for this comment. Very helpful, we have revised our abstract accordingly. Please see lines 55-59

Awareness and knowledge about FGS among MPMS and HCPs were poor in Anambra, Nigeria. It is therefore important to invest in innovative methods of building capacity of MPMS and HCPs, with complementary provision of necessary diagnostics to perform colposcopy, as well as competence to diagnose pathognomonic lesions using diagnostic atlas or Artificial Intelligence (AI).

**Comment 17:** Page 6 Line 66-67: FGS knowledge is measured but not for schistosomiasis?

**Response 17:** Yes we asked the students about their awareness on both diseases (FGS and Schistosomiasis), but we asked the HCPs about their knowledge on both disease. We have revised the section accordingly.

**Comment 18:** Page 7 Line 78: Which region?

**Response 18:** Thank you very much for your comment. We have revised the manuscript accordingly.

Please see line 86

Two major Schistosoma species are common in Africa,

**Comment 19:** Page 7 Line 94: please merge references

**Response 19:** Thank you very much for your comment. We have revised the manuscript accordingly.

**Comment 20:** Page 8 Line 104: Please check all sections and merge references

**Response 20:** Thank you very much for your comment. We have revised the manuscript accordingly.

**Comment 21:** Page 8 Line 105: The latest terminology is homogenous yellow patches (remove sandy)

**Response 21:** Thank you very much for your comment. We have revised the manuscript accordingly

**Comment 22:** Page 9 Line 111: Foreigners from where? Do you mean from non-endemic countries?

**Response 22:** Thank you very much for your comment. We have revised the manuscript accordingly.

Please see line 127

Foreigners from non-endemic countries who visit infested freshwater bodies are also at risk [14].

**Comment 23:** Page 9 Line 114: I would remove accommodating

**Response 23:** Thank you very much for your comment. We have removed the word accomodating

**Comment 24:** Page 9, Line 117-118: This is not clear, please rephrase

**Response 24:** Thank you very much for your comment. We have revised the manuscript accordingly.

Please see line 131-134

Interestingly, the preliminary findings from both states have spurred the interest of healthcare professionals and researchers to understand the epidemiology of FGS and also develop protocols aimed at investigating FGS endemicity in other regions in the country.

**Comment 25:** Page 9, Line 122: Training gap

**Response 25:** Thank you very much for your comment. We have revised the manuscript accordingly.

**Comment 26:** Page 9, Line 122-123: Remove hence resulting in

**Response 26:** Thank you very much for your comment. We have revised the manuscript accordingly.

Line 139-141

These training gaps might contribute to misdiagnosis/classification of clinical manifestations due to FGS, resulting in stigmatization, mental stress, and social exclusion of young females

**Comment 27:** Page 10 Line 124-126: Please specify the students you are referring to, and the expertise of the health care professionals

**Response 27:** Thank you very much for your comment. We have revised the manuscript accordingly.

Line 142-148

We therefore aim to understand the knowledge and perception of FGS among medical and paramedical students who subsequently transform into HCPs, as well as expertise of current HCPs who are saddled with the responsibility of delivering care to FGS patients. These findings would be useful to support the call for local investments, collaborations and innovations, when deliberations towards controlling FGS are been mapped out by schistosomiasis control programmers, researchers and policy makers in the state, and Nigeria at large.

**Comment 28:** Page 10 Line 128-129: you can rephrase this and take to discussion

**Response 28:** Thank you very much for your comment. We have revised the manuscript accordingly.

**Comment 29:** Page 10 Line 136-137: This statement seems to be misplaced and incomplete

**Response 29:** Thank you very much for your comment. We have revised the manuscript accordingly.

**Please see line 152-157**

**Ethics Statement and considerations**

This study received ethical approval (COOUTH/CMAC/ETH.C/Vol.1/FN:04/0117) from the ethical review board of Chukwuemeka Odumegwu Ojukwu University Teaching Hospital. Consent was sought from study participants prior to data collection, and only participants who provided written informed consent were recruited. Unique identifiers were used in place of participants’ name to ensure anonymity, while a password-protected database was used to archive all datasets to ensure confidentiality.

**Comment 30:** Page 11 Line 155: Again, please be specific

**Response 30:** Thank you very much for your comment. We agree perfectly with you, and we have now revised the methodology section entirely to show the details of our sampling approach and clearly define the population. We appreciate the reviewer for this comment., which significantly added value to our manuscript

Please see line 173-207

**Study design, mobilization, recruitment of study participants, and data collection**

This study was conceptualized following the guidelines of Bridges to Development, and The Geneva Learning Foundation on the FGS Accelerated Scale Together (FAST) Package. The study was designed as a cross-sectional survey to assess the perception and expertise of female MPMS and HCPs using quantitative methods. Two electronic questionnaires were developed during the First Scholar Workshop on Female Genital Schistosomiasis (FGS) (<https://www.learning.foundation/female-genital-schistosomiasis>) and the First FGS Impact Accelerator meeting (<https://www.learning.foundation/fgs>) held between May and October 2021. Questionnaires were pilot tested among two external supervisors and 10 female students in Anambra, before deploying it for field use in October 2021. However, physical interactions were impossible in October, because of COVID-19 lockdown orders and closure of schools during the strike actions by academic unions across all government -owned universities in the country. As such, we employed electronic mode of enrollment and administration of questionnaires. We recruited medical and para-medical female students enrolled in Nnamdi Azikiwe University in Awka. This university was purposively selected because it is government-owned, and with the largest enrollment due to its low tuition fee when compared to other universities in the state.

A minimum of 500 medical and paramedical female students from the three faculties; biosciences, medical sciences and pharmacy, were targeted for recruitment. These students were approached through the headship of faculty-based student unions, and subsequently invited to participate in the study through personal invitation letters sent to their institutional email. Follow-up recruitments were done through dedicated WhatsApp and Telegram platforms. An electronic packet that contains a brief introductory note about the project, a question to seek consent, and the main questionnaire were sent across the platforms. Only submissions of those who completed the consent questionnaire were included in the final analysis.

Furthermore, we targeted 50 of the 100 health care practitioners who operate at the State level, and are members of the schistosomiasis control committee. These healthcare practitioners (HCPs) were interviewed physically during one of the routine stakeholder meetings organized by the Anambra State Schistosomiasis control and elimination program. Questionnaires were self-administered to consenting stakeholders before the commencement of the meeting. Considering the literacy status of participants (MPMS and HCPs), all questionnaires were designed and administered in English language.

**Comment 31:** Page 11 Line 161-164: This can be two sentences and please use past tense

**Response 31:** Thank you very much for your comment. We have revised the manuscript accordingly. Infact, we reworked the entire section as we have highlighted above in response 30. Please see line 173-207

**Comment 32:** Page 11 Line 167-168: How did you get the contact information such as email addresses

**Response 32:** Thank you very much for your comment. We have revised the manuscript accordingly. We got the emails through the headship of faculty-based student unions. Infact, we reworked the entire section as we have highlighted above in response 30. Please see line 173-207

**Comment 33:** Page 11 Line 170-171: Please rephrase to be clear

**Response 33:** Thank you very much for your comment. We have revised the manuscript accordingly. Please see line 204-205

Questionnaires were self-administered to consenting stakeholders before the commencement of the meeting.

**Comment 34:** Page 11 Line 171-172: Was is the same questionnaire for students and health workers

**Response 34:** Thank you very much for your comments. The questionnaires were different. To provide more clarity, we have added a new sub-section “Study indicators”. Here we explained in details the indicators used, and also added a supplementary file to the tools.

Please see line 208-225 for the study indicators

**Study indicators**

We used two separate tools (See S1 File) and (See S2 File) to document responses from female MPMS and HCPs, respectively. For HCPs, demographic information including age, gender, profession, year of practice and place of work were documented. In addition, we collected information on HCPs expertise on a range of symptoms/condition that overlaps with FGS pathology. These included; (i) if the practitioner performs pelvic examination, (ii) treat venereal diseases, (ii) lower abdominal pain, (iv) dysuria or (v) hematuria. Knowledge of HCPs about schistosomiasis and FGS were also documented, with emphasis on (i) awareness, (ii) source of information, (iii) transmission routes, (iv) preventive practices, (v) treatment choices with emphasis on pregnant women, and (vi) dosage requirements for children and adult. HCPs were also asked if FGS patients should be quarantined and if care commodities for FGS patients are available in their place of work (See S1 File)

Similarly for female MPMS, we collected information on age, religion, parents’ occupation and income. In addition, knowledge about schistosomiasis and FGS were documented, with emphasis on awareness and source of information. We also documented if MPMS (i) have ever been treated with praziquantel, (ii) had contact with freshwater, or (iii) had experienced any of the following symptoms that overlaps with FGS pathology; hematuria, painful urination, lower abdominal pain and venereal diseases (See S2 File).

**Comment 35**: Page 12 Line 135: You referred a lot to "significantly poor, significant lack of awareness and knowledge", it is not clear how the knowledge was measured and categorized as such

**Response 35:** Thank you very much for your comment. We have revised the manuscript accordingly and avoided the use of the word significantly poor/lack, rather we used the word “low” and “high”

**Comment 36**: Page 12 Line 186-187: Consider using comma after 650 and professionals

**Response 36:** Thank you very much for your comment. We have revised the manuscript accordingly

**Comment 37:** Page 12 Line 189: It was not clear if this was included in the questionnaire

**Response 37:** Yes it was included in the questionnaire, we have attached copies of the questionnaire and also included a section “study indicators” that described the questionnaire in details. Please see line 208-225 for the study indicators

**Comment 38:** Page 12 Line 194-195: Past tense

**Response 38:** Thank you very much for your comment. We have revised the manuscript accordingly

**Comment 39:** Page 12 Line 196: Is this 87% of the practitioners with less than a decade, or 87% of all the health care professionals

**Response 39:** Thank you very much for your comment. We have revised the manuscript accordingly. Please see line 258-260

About half of the practitioners have spent less than a decade in their current position. Also, 87% of the practitioners work in a government-owned facility. However, only 46% of them have experience conducting pelvic examinations

**Comment 40:** Page 12 Line 197: I would refer to the Table at the beginning of the paragraph

**Response 40:** Thank you very much for your comment. We have revised the manuscript accordingly.

**Comment 41:** Page 13 Line 205: If this is appearing for the first time, please write in full

**Response 41:** Thank you very much for your comment. We have revised the manuscript accordingly.

**Comment 42:** Page 13 Line 205: Please remove the comma

**Response 42:** Thank you very much for your comment. We have revised the manuscript accordingly.

**Comment 43:** Page 13 Line 206: Past tense

**Response 43:** Thank you very much for your comment. We have revised the manuscript accordingly. Please see line 270

However, only 246 (41.9%) of the participants have heard about FGS

**Comment 44:** Page 13 Line 209: I would refer to the Figure at the beginning of the paragraph

**Response 44:** Thank you very much for your comment. We have revised the manuscript accordingly.

**Comment 45:** Page 14 Line 232-235: It is not clear if these % are referring to the practitioners or patients. Please rephrase

**Response 45:** The % here refers to the practitioners. We have revised the manuscript accordingly.

Please see line 317-320;

Table 3 show the prevalence of schistosomiasis suspicion among practitioners who offer care. Majority of the practitioners suspected schistosomiasis during their diagnosis of dysuria (60.6%), hematuria (58.5%), significant lower abdominal pain (56.1%) and venereal diseases (53.7%).

**Comment 46:** Page 15 Line 241: Table 2: You mentioned above that the 65 health care professionals recruited varied in specialty, but here you have a total of 65 that can treat. Please correct or clarify

**Response 46:** Thank you very much for your comment. We have revised the manuscript accordingly by removing the 65 to allow confusion. The NT which refers to the number of practitioners who treat one form of clinical condition was retained. Pease see Table 3 (because we added a new table). So the previous table 2 is now table 3.

**Comment 47:** Page 15 Line 246- 247: Please remove the comma and rephrase

**Response 47:** Thank you very much for your comment. We have revised the manuscript accordingly. Please see Line 330

About 43% of the practitioners were uncertain if they should quarantine patients with FGS.

**Comment 48:** Page 15 Line 250-251: Please check the use of past tense in the entire manuscript

**Response 48:** Thank you very much for your comment. We have revised the manuscript accordingly. Please see line 334-335.

In addition, about 35% of them do not know the dosage requirements for praziquantel tablets (Table 3).

**Comment 49:** Page 15 Line 264-266: Is this a new category of participants in addition to the students and health care professionals? this was not mentioned in the objective and methodology. Or is this information obtained from the interview with the practitioners? Please clarify and rephrase the paragraph

**Response 49:** Thank you for this comment. We apologize for the mix-up, and absolutely agree with the reviewer. The respondents reported here on Line 291, are sub-set of the 587 female students in this study. From our analysis, a sub-set of the 587 students reported they had hematuria (n=31), painful urination (n=83), lower abdominal pain (n=187) and venereal diseases (n=83), and that they recently sought care from medical practitioners. As such, their data were separated for an in-depth analysis to document their experience with the practitioner who cared for them.

We have revised the manuscript accordingly. Please see line 345-351

**Participants' experiences during routine healthcare service on FGS-related symptoms**

Of the 587 female MPMS, 83 presented a case of painful urination to the health care center, and only 22 (26.5%) of them were asked if they had recent contact with fresh water bodies (p=0.458). Similarly, only 12 (38.7%) of the 31 students who presented hematuria were asked (p=0.035). In addition, only 44 (23.5%) of 187 who presented lower abdominal pain were asked (p=0.786), and only 31 (37.3%) of the 83 residents who presented symptoms of venereal diseases were asked (p=0.000) (Table 5).

Also, see line 411-414

Largely, 60-70% of the female students who reported painful urination, hematuria, lower abdominal pain, and venereal diseases also affirmed that healthcare professionals had never asked them about their history of contact with rivers when they sought medical care.

**Comment 50**: Page 15 Line 268: Table 5: what is NE

**Response 50:** Thank you very much for your comment. We have revised the manuscript accordingly

**REVIEWER 3:**

**General comment:** The study gives an overview of knowledge of health care professionals and students in a region in Nigeria where FGS is endemic. It would be useful to stress more clearly how difficult it is to identify FGS and to address the syndromic approach as a possible manner to reach more persons which might have FGS. It would be useful to identify if the mentioned university the curriculum includes FGS or schistosomiasis. please refine your study population as mentioned above.

**Response: We** thank the reviewer for the great deal of effort expended in reviewing this manuscript and pointing out very critical points and areas that needed attention. Your contributions are well appreciated.

**Comment 1:** Objectives of the study are clearly stated.

**Response 1:** Thank you very much for your comment

**Comment 2:** Study design is appropriate.

**Response 2:** Thank you very much for your comment

**Comment 3:** Study population is doubtful as only female students were included. Among the health workers also personel not qualified to provide FGS care were included such academic reserachers or lab personnel. Please exclude those persons from your analysis.

**Response 3:** Thank you very much for your comment. We agree perfectly with you, and we have now revised the methodology section entirely to show the details of our sampling approach. We appreciate the reviewer for this comment., which significantly added value to our manuscript. In addition, in the demography analysis, we included all those who identified as HCPs (even if they were not qualified to provide FGS care), because they are members of schistosomiasis control committee, and are saddled with the responsibility of guiding the actions and policies of the committee as regards schistosomiasis control. As such, we consider it is important to understand their perception about FGS. However, for questions related to FGS care (Table 2 and Table 3), we excluded the HCPs who do not provide FGS care in the analysis. Thank you for your direction.

Please see line 173-186 for the improved study methodology.

**Study design, mobilization, recruitment of study participants, and data collection**

This study was conceptualized following the guidelines of Bridges to Development, and The Geneva Learning Foundation on the FGS Accelerated Scale Together (FAST) Package. The study was designed as a cross-sectional survey to assess the perception and expertise of female MPMS and HCPs using quantitative methods. Two electronic questionnaires were developed during the First Scholar Workshop on Female Genital Schistosomiasis (FGS) (<https://www.learning.foundation/female-genital-schistosomiasis>) and the First FGS Impact Accelerator meeting (<https://www.learning.foundation/fgs>) held between May and October 2021. Questionnaires were pilot tested among two external supervisors and 10 female students in Anambra, before deploying it for field use in October 2021. However, physical interactions were impossible in October, because of COVID-19 lockdown orders and closure of schools during the strike actions by academic unions across all government -owned universities in the country. As such, we employed electronic mode of enrollment and administration of questionnaires. We recruited medical and para-medical female students enrolled in Nnamdi Azikiwe University in Awka. This university was purposively selected because it is government-owned, and with the largest enrollment due to its low tuition fee when compared to other universities in the state.

A minimum of 500 medical and paramedical female students from the three faculties; biosciences, medical sciences and pharmacy, were targeted for recruitment. These students were approached through the headship of faculty-based student unions, and subsequently invited to participate in the study through personal invitation letters sent to their institutional email. Follow-up recruitments were done through dedicated WhatsApp and Telegram platforms. An electronic packet that contains a brief introductory note about the project, a question to seek consent, and the main questionnaire were sent across the platforms. Only submissions of those who completed the consent questionnaire were included in the final analysis.

Furthermore, we targeted 50 of the 100 health care practitioners who operate at the State level, and are members of the schistosomiasis control committee. These healthcare practitioners (HCPs) were interviewed physically during one of the routine stakeholder meetings organized by the Anambra State Schistosomiasis control and elimination program. Questionnaires were self-administered to consenting stakeholders before the commencement of the meeting. Considering the literacy status of participants (MPMS and HCPs), all questionnaires were designed and administered in English language.

**Comment 4:** Please provide information on the sample size and regarding power calculation. If not done please provide a reason for it.

**Response 4:** Thank you very much for your comment Please see the revised lines	173-207 (response above), where we described in details how we arrived at our sample size.

A minimum of 500 medical and paramedical female students from the three faculties; biosciences, medical sciences and pharmacy, were targeted for recruitment. These students were approached through the headship of faculty-based student unions, and subsequently invited to participate in the study through personal invitation letters sent to their institutional email. Follow-up recruitments were done through dedicated WhatsApp and Telegram platforms. An electronic packet that contains a brief introductory note about the project, a question to seek consent, and the main questionnaire were sent across the platforms. Only submissions of those who completed the consent questionnaire were included in the final analysis.

Furthermore, we targeted 50 of the 100 health care practitioners who operate at the State level, and are members of the schistosomiasis control committee. These healthcare practitioners (HCPs) were interviewed physically during one of the routine stakeholder meetings organized by the Anambra State Schistosomiasis control and elimination program. Questionnaires were self-administered to consenting stakeholders before the commencement of the meeting. Considering the literacy status of participants (MPMS and HCPs), all questionnaires were designed and administered in English language.

**Comment 5:** Statistical analysis is appropriate for the reserach questions, futher it could be adresses which are the determinants of correct management of FGS using regression analysis.

**Response 5:** Thank you very much for your comment. We have performed a regression analysis (please see table 2, line 311) to model the relationship between year of study (for students), year of practice and expertise (for HCPs) on the knowledge about FGS and Schistosomiasis [as separate outcome variables]. We found that students in the upper years of study had very higher odds of been more informed for schistosomiasis, but we didn’t find any association with FGS, and the HCPs year of practice and expertise was also not associated with knowledge of either disease. We believe the very small sample size of HCPs recruited (n=65), might have influenced the outcome of this analysis. However, we thought (in future studies) of expanding the sampling frame to other healthcare professionals at regional level rather than the state level as we have done in this present study). We really appreciate the reviewer for pointing us towards the direction of an additional analysis.

**Comment 6**: the results mach the analysis plan. Results are clearly presented. Tables and images quality is ok.

**Response 6:** Thank you very much for your comment.

**Comment 7:** Conclusion support the data presention. However, it should be stated more clearly that part of the mentioned diagnostic facilities are not suitable for the diagnosis of FGS which required the tools and the competences to perform colposcopy as well as the competence to diagnose pathognomic leasion, eventually with the help of a diagnostic atlas or with AI based App support.

**Response 7:** Thank you very much for your comment. We have revised the manuscript accordingly

**Line 447-456**

Our study has highlighted the low level of awareness about FGS among university students and healthcare professionals, as well as poor expertise of healthcare professionals towards FGS care. Our findings show the probable effect of these gaps on underdiagnosis, misdiagnosis, and/or lack of or inappropriate treatment for FGS patients. It is therefore important to develop and incorporate FGS training materials in training curricula of university students and training programs of health professionals during routine ongoing schistosomiasis control programs. Furthermore, this training should be complemented with provision of appropriate medical diagnostics for schistosomiasis and FGS, and on-site competence building towards performing colposcopy, and diagnosing pathogenic lesions using diagnostic atlas or Artificial Intelligence (AI) applications.

**Comment 8**: The limitiations of the analysis should adress the sample selection and stress further the distinction between the diagnosis of FGS and urinary schisto. The autors adress how the data could advance the understanding of public health management.

**Response 8:** Thank you very much for your comment. We have revised the manuscript accordingly

Please see line 459-469

**Limitations of the study**

This study was conducted during the COVID-19 pandemic with considerable restrictions on movements, hence we employed an electronic mode of data collection for the students recruited. In addition, we assessed participants’ awareness/knowledge about schistosomiasis and FGS using a binary outcome, hence we cannot ascertain if respondents know the true definitions and conditions of the diseases. Thirdly, some of the medical and para-medical students affirmed they learnt about schistosomiasis from school, and our quantitative methods could not probe further the exact source of knowledge. We therefore understand their responses may be subject to recall bias. Finally, we recruited a very small number of HCPs (n=65) who operate at the State level in the schistosomiasis control program, and some of the specializations were underrepresented. We believe a more robust evaluation of practitioner’s expertise at regional and community level may be very useful.

**Comment 9:** **Abstract; Methodology:** Please explain why only female students were interviewed., Why should only female students be aware of the disease

**Response 9:** Thank you very much for this comment. We actually agree we could have expanded our participants frame to male students, and there is no justification for such omission. However, FGS is gaining considerable attention in the country at the moment, and at the point of conceptualization of this study, our interest (in addition to profiling the awareness and knowledge) was to particularly explore experiences of females with probable symptoms of FGS when they sought medical care. This we thought would also provide supportive evidence to t the assessment of expertise among health care professional

**Comment 10; Line 46:** Please specify percentages

**Response 10:** Thank you very much for this comment. We have updated the abstract sections with results from the analysis you suggested. We must thank the reviewer for pointing our direction to this.

**Please Line 43-**53

Over half of the students recruited; 54.2% for schistosomiasis and 58.1% for FGS, were not aware of the disease. However, Students in the 2^nd^ (OR: 1.66, 95% CI: 1.0, 2.7), 4^th^ (OR: 1.97, 95% CI: 1.2, 3.2), and 6^th^ (OR: 5.05, 95% CI: 0.4, 5.5) year of study have higher likelihoods of been more informed about schistosomiasis. For HCPs, we observed a contrastingly high knowledge about schistosomiasis (96.9%), but low knowledge about FGS (61.9%). Knowledge was also not associated with year of practice and expertise (p > 0.05). A considerable proportion (>40%) of the HCPs never suspected schistosomiasis during routine clinical diagnosis of patients who presented probable FGS symptoms (p < 0.05). Similarly, only 20% were certain about the use of praziquantel for treating FGS, and about 35% were uncertain of the eligibility criteria and dosage regimens. Commodities for managing FGS were also largely unavailable in about 39% of the health facilities where the HCPs operate.

**Comment 11: Line 48:** Please specify percentages

**Response 11: Please Line 43-**53

Over half of the students recruited; 54.2% for schistosomiasis and 58.1% for FGS, were not aware of the disease. However, Students in the 2^nd^ (OR: 1.66, 95% CI: 1.0, 2.7), 4^th^ (OR: 1.97, 95% CI: 1.2, 3.2), and 6^th^ (OR: 5.05, 95% CI: 0.4, 5.5) year of study have higher likelihoods of been more informed about schistosomiasis. For HCPs, we observed a contrastingly high knowledge about schistosomiasis (96.9%), but low knowledge about FGS (61.9%). Knowledge was also not associated with year of practice and expertise (p > 0.05). A considerable proportion (>40%) of the HCPs never suspected schistosomiasis during routine clinical diagnosis of patients who presented probable FGS symptoms (p < 0.05). Similarly, only 20% were certain about the use of praziquantel for treating FGS, and about 35% were uncertain of the eligibility criteria and dosage regimens. Commodities for managing FGS were also largely unavailable in about 39% of the health facilities where the HCPs operate.

**Comment 12: Authors summary:** please provide a broader picture of disease, do no repeat the abstract

**Response 12:** Thank you very much for your comment. We have revised the manuscript accordingly. Please see line 64-80

Female genital schistosomiasis (FGS) is a neglected disease that is often mistaken for other sexually transmitted infections. The disease has thus received little attention at the primary health care unit until recent. To support the emerging momentum been built towards controlling this disease, we conducted this study to understand the knowledge and perception of FGS among medical and paramedical students (MPMS) who subsequently transform into HCPs, as well as expertise of current HCPs who are saddled with the responsibility of delivering care to FGS patients. 587 MPMS and 65 HCPs were interviewed in Anambra State, using well designed questionnaires. Our findings show that majority of the MPMS were not aware of schistosomiasis and FGS, but students in the 6^th^ year of study have higher chances of been informed about schistosomiasis. However, almost all the HCPs were aware of schistosomiasis, but FGS awareness was very low. The HCPs also lack the capacity and resources to diagnose and manage FGS. This significant gap in knowledge, capacity and resources calls for development of FGS training materials, and its subsequent incorporation in curricula of medical students, and training of HCPs during routine schistosomiasis control programs. This should be complemented with the provision of necessary diagnostics to perform colposcopy, as well as building competence to diagnose pathognomonic lesions using diagnostic atlas or Artificial Intelligence (AI).

**Comment 13:** **Line 80-83:** please explain better on which factors the pathology depends on.

**Response 13:** Thank you very much for your comment. We have explained between lines 87-91 that the pathologies are dependent on migration of the worms through the organs and body tissues, and inflammatory responses to the presence of the eggs laid by the adult worms

Please see lines 87-91

The pathologies associated with both species vary depending on several factors, which are not limited to the severity of infection (duration and intensity), migration of the worms through the organs and body tissues, and inflammatory responses to the presence of the eggs laid by the adult worms [3].

**Comment 14:** **Line 84-85:** please explain further what the long-term consequences of Schistosoma mansoni can be, beyond hepatosplenomegaly

**Response 14:** Thank you very much for your comment. We have revised the manuscript accordingly.

**Please see lines 93-96**

The inflammatory reactions in the liver could lead to hepatosplenic schistosomiasis, which is a key feature of chronic infection. The fibrotic lesions produced by this inflammation could lead to liver cirrhosis that progressively occludes the portal system giving rise to portal hypertension, which could eventually lead to death [5].

Comment 15: **Line 85** : remove however.

**Response 15:** Thank you very much for your comment. We have revised the manuscript accordingly

Comment 16: **Line 89:** please explain in detail the pathophysiology of FGS

**Response 16:** We explained this already in the second paragraph between lines 103-113

Comment 17: **Line: 93:** to my knowledge the sole presence of Schistosoma DNA does not define FGS, without any compatible clinical symptoms.

**Response 17:** Thank you very much for your comment, we have replaced the word “or” with “with” to address this comment. See line 103-105

FGS is a clinical condition used to describe the presence of trapped *S. haematobium* eggs, DNA, with characteristic clinical changes specifically in the genital tract of affected women, regardless of whether or not, the eggs are present in the urinary tract [5,6].

Comment 18: **Line 101**: please describe also pregnancy related problems.

**Response 18:** Thank you very much for your comment, we have revised the manuscript. Please see line 113-115

Furthermore, for reproductive women, FGS is associated with poor pregnancy outcome such as stillbirth, spontaneous abortion, and ectopic pregnancy [10]

Comment 19: **Line 104:** please provide Information on psychosocial consequences of FGS

**Response 19:** Thank you very much for your comment. We have revised the manuscript accordingly. Please see Lines 113-118

These reproductive health consequences (fertility issues and poor pregnancy outcome) often lead to discrimination, depression, marital discord and social stigma, particularly when FGS is mistaken for other sexually transmitted infections (STI) [11,12,13]

Comment 20: **Line 119:** please explain what Bridges for development is

**Response 20:** Thank you very much for your comment. We have revised the manuscript accordingly. Please see Lines 134-137

This interest has been accompanied by investment in capacity building of health care professionals from Bridges to Development, a nonprofit and tax-exempt organization in Switzerland and the United State of America, focused on advancing economic and social well-being of women and girls [17].

Comment 21: Line 122: this is an assumption. “Gaps in training might contribute” …

**Response 21: Response 20:** Thank you very much for your comment. We have revised the manuscript accordingly. Please see Lines 139-142

These training gaps might contribute to misdiagnosis/classification of clinical manifestations due to FGS, resulting in stigmatization, mental stress, and social exclusion of young females.

Comment 22: Line: 124: cite appropriate literature here

**Response 22:** Thank you very much for your comment. We have revised the manuscript accordingly. Please see Lines 142

Comment 23: Line 125: please provide a rationale why only female students are of interest.

**Response 23:** Thank you for this comment. We have spent sufficient time from line 118 building the rationale on why we focused on females, considering the burden of FGS amongst them the associated consequences. Also, at the conceptualization of this study, our interest (in addition to profiling the awareness and knowledge) was to particularly explore experiences of females with probable symptoms of FGS and their experience when they sought medical care. This we thought would also provide supportive evidence to t the assessment of expertise among health care professional

Comment 24: please provide information on how to diagnose FGS, questions in the methodology focus on the diagnosis of urinary schistosomiasis not FGS

**Response 24:** We agree with you absolutely that our questionnaire didn’t focus on how to diagnose FGS (we didn’t include questions that assess if the practitioners could perform coloscopy, or identify lesions etc.). Rather our interest in this first manuscript was to investigate the awareness and knowledge towards schistosomiasis and FGS, and explore the expertise of practitioners, especially if they could suspect potential risk factors (i.e., schistosomiasis, water contact behaviors) among patients who reports probable symptoms of FGS. We thank the reviewer for this comment and would consider the inclusion of questions related to diagnosing FGS in a more robust study (where we plan to recruit and assess health care practitioners who operates at the regional and communal level).

**Comment 26:** Line 138: please provide more details on data safety. Where did you safe electronic data?

**Response 27:** Thank you very much for your comment. We have revised the manuscript accordingly. Please see the lines detailing where the electronic data were saved. Only the PI has access to such electronic data

**Line 155-157:** Unique identifiers were used in place of participants’ name to ensure anonymity, while a password-protected database was used to archive all datasets to ensure confidentiality.

**Line 228-**229: Data collected were imported from password protected online repository into R studio for analysis.

**Comment 27: Results:** Line 192: the group of health care professionals seems very heterogeneous, it seems unlikely to gain overview of knowledge from this group, why should a lab scientist be aware of treatment and diagnostic procedures on FGS. This is beyond the scope of his profession. Please exclude all profession which to not have patient contact and cannot potentially be confronted with patients having FGS. Or provide a specific reason to include them in your sample. It would be interesting to see if there are knowledge difference between medical personnel and non-medical personnel providing patient care

**Response 27:** Thank you very much for your comment. We agree perfectly with you, and we have now revised the methodology section entirely to show the details of our sampling approach. We appreciate the reviewer for this comment., which significantly added value to our manuscript. In addition, in the demography analysis, we included all those who identified as HCPs (even if they were not qualified to provide FGS care), because they are members of schistosomiasis control committee, and are saddled with the responsibility of guiding the actions and policies of the committee as regards schistosomiasis control at the State level. As such, it is important to understand their perception about FGS. However, for questions related to care (Table 3), we excluded all those HCPs, who do not provide care. We have also made additional regression analysis (Table 2) to show if there are knowledge differences between the categories of HCPs, and our results show no knowledge difference.

**Comment 28:** Line: 197: this is logical if you include professional groups which do not work in clinical care

**Response 28:** and it also becomes worrisome, because they are members of schistosomiasis control committee, and are saddled with the responsibility of guiding the actions and policies of the committee as regards schistosomiasis control at the State level. These gaps show the need to build capacity across board (care providers and schistosomiasis technical committee who in most cases comprises of personnel who are not medics).

**Comment 29:** Line 201: please stratify your results according to years of study in medicine. It might be that younger students know less simply because they did to touch up on the topic yet.

**Response 29:** Thank you very much for your comment. We have performed a regression analysis to model the relationship between year of study (for students), year of practice and expertise (for HCPs) on the knowledge about FGS and Schistosomiasis [as separate outcome variables]. Please see the new table 2. We found that students in the upper years of study had very higher odds of been more informed for schistosomiasis, but we didn’t find any association with FGS, and the HCPs year of practice and expertise was also not associated with knowledge of either disease. We believe the very small sample size of HCPs recruited (n=65), might have influenced the outcome of this analysis. However, we thought (in future studies) of expanding the sampling frame to other healthcare professionals at regional level rather than the state level as we have done in this present study). We really appreciate the reviewer for pointing us towards the direction of an additional analysis.

**Comment 30:** Line 239: table 2 show symptoms not diseases, Please specify if these cases you asked for FGS suspicion or schistosomiasis suspicion.

**Response 30:** With the addition of a new table, this table is now Table 3. Yes, on this table we worked with data of practitioners who offer care to patients with probable FGS symptoms. And we asked if they ever suspected schistosomiasis during their diagnosis of the listed probable FGS symptoms.

**Comment 31:** Line 252: please provide clear information that urinalysis test strips and schisto detection kits do are commodities to provide diagnosis for urinary schisto but not for FGS. – This information needs to be placed in the background.

**Response 31:** Thank you very much for your comment. We have revised the manuscript accordingly.

**Comment 32:** Line 251: Table 3 please provide this information only for personal having patient contact N 41

**Response 32:** Thank you very much for your comment. We have performed the new analysis with the data of the 41 HCPs who offer care. Please see new table 4

**Comment 33:** Line 266: Table 5 did these patients presented venereal disease or symptoms compatible with venereal disease which could be attributed to FGS.

**Response 33:** Our questionnaire didn’t probe further into the specific symptoms of the venereal disease, rather it asked only if the study participants had been treated of venereal infection recently, and the data of these participants were cross-tabulated with their experience with a medical practitioner when they sought medical care. We were particularly interested in identifying if the health care practitioner asked about the most common risk factor of schistosomiasis (contact with fresh water).

**Comment 34:** Discussion: Line 293: add Ref. Schuster et al <https://journals.plos.org/plosntds/article?id=10.1371/journal.pntd.0010901>

**Response 34:** Many thanks for the reference. This has now been cited.

**Comment 35:** Line 295: wording

**Response 35:** Thank you very much for your comment. We have revised the manuscript

**Comment 36:** Line 299. This is wrong, some of the interviewed HCP are not engaged in schistosomiasis control activities.

**Response 36:** Thank you for your comment. However, all the HCPs we interviewed in this study are all engaged in schistosomiasis control program, but at different capacity. In the demography section of this manuscript, we have included all the specializations, as such we have those who are medic, and those who are non-medics. But they are all members of schistosomiasis control committee, and are saddled with the responsibility of guiding the actions and policies of the committee as regards schistosomiasis control at the State level. As such, we consider it is important to understand their perception about FGS.

**Comment 37:** Line 307: would it be possible to check of Schistosomiasis and FGS is part of the curriculum at the medical university? It might be useful to differentiate if it is taught but not thought well or if it is not taught. Please stratify your results by years of study. or if not possible by age. ( see below in methods section)

**Response 37:** Thank you very much for your comment. Schistosomiasis is taught as part of the content in the basic biology and parasitology course to first year science students in Nigeria, and subsequently later in their 3^rd^ year. However, the content varied per tutor (i.e., not regularized) and the extent to which the contents are properly taught is unknown. Based on your suggestion, we have performed a regression analysis to model the relationship between year of study (for students), year of practice and expertise (for HCPs) on the knowledge about FGS and Schistosomiasis [as separate outcome variables]. Please see table 2. We found that students in the upper years of study had very higher odds of been more informed for schistosomiasis, but we didn’t find any association with FGS, and the HCPs year of practice and expertise was also not associated with knowledge of either disease. We believe the very small sample size of HCPs recruited (n=65), might have influenced the outcome of this analysis. However, we thought (in future studies) of expanding the sampling frame to other healthcare professionals at regional level rather than the state level as we have done in this present study). We really appreciate the reviewer for pointing us towards the direction of an additional analysis

**Comment 38:** Line 319. Reported symptoms in line with venereal infection.

**Response 38:** Thank you very much for your comment. We have revised the comment accordingly. Please see line 413

**Comment 39:** line 329: urinary filtration kits and kato katz does not allow to diagnose FGs but urinary or intestinal schistosomiasis. Urinary schistosomiasis is often associated with FGS. Solely the possibility to perform colposcopy together with the needed diagnostic competencies would allow to diagnose FGS. Therefore, it should be stressed that material and competences perform colposcopy as well the diagnostic knowledge to identify pathognomonic lesions on the cervix should be promoted.

**Response 39:** Thank you very much for your comment. We have revised the comment accordingly. Please see line 418-425

Furthermore, the lack of commodities such as Kato Katz and urine filtration kits which are important diagnostic materials for urogenital and intestinal schistosomiasis is also of concern, as urogenital schistosomiasis is often associated with FGS. This is further worsened by the lack of materials for colposcopy and competence to identify pathognomonic lesions which are necessary tools for diagnosing FGS. It is therefore important to complement discussion around increasing awareness and knowledge of health care professionals, with considerable investments and provision of adequate commodities for detecting and treating schistosomiasis and FGS.

And Line 437-443

It is, therefore, important to highlight that currently developed tools can be adapted for local pilot among healthcare professionals in the study area, to improve health workforce capacity and contribute to alleviating the neglect of FGS. This training should also be complemented with provision of appropriate diagnostics and in-field support towards building the competence of qualified health care professionals to perform colposcopy, as well as diagnose pathogenic lesions with the help of a diagnostic atlas or with Artificial Intelligence (AI) based App support. [L]

**Comment 40:** Diagnostic helpers such as and diagnostic atlas with pathognomic images and Ai based application to support health workers in the diagnosis of FGS should be mentioned (<https://ieeexplore.ieee.org/document/9874491>)

**Response 40:** Thank you very much for this wonderful citation. We have revised the section accordingly and added this reference. Please see line 445

This training should also be complemented with provision of appropriate diagnostics and in-field support towards building the competence of qualified health care professionals to perform colposcopy, as well as diagnose pathogenic lesions with the help of a diagnostic atlas or with Artificial Intelligence (AI) based App support[47]

**Comment 41: Limitations:** Address the fact that only female students were interviewed

**Response41:** Thank you very much for this comment. We really appreciate the efforts of the reviewer

**Please see Line 459-469:**

This study was conducted during the COVID-19 pandemic with considerable restrictions on movements, hence we employed an electronic mode of data collection for the students recruited. In addition, we assessed participants’ awareness/knowledge about schistosomiasis and FGS using a binary outcome, hence we cannot ascertain if respondents know the true definitions and conditions of the diseases. Thirdly, some of the medical and para-medical students affirmed they learnt about schistosomiasis from school, and our quantitative methods could not probe further the exact source of knowledge. We therefore understand their responses may be subject to recall bias. Finally, we recruited a very small number of HCPs (n=65) who operate at the State level in the schistosomiasis control program, and some of the specializations were underrepresented. We believe a more robust evaluation of practitioner’s expertise at regional and community level may be very useful
