# Supplementary material for: Profiling the knowledge of female medical/para-medical students, and expertise of health care professionals on female genital schistosomiasis in Anambra, South Eastern Nigeria
Source: PLoS Negl Trop Dis. 2023 Feb 16;17(2):e0011132. doi: 10.1371/journal.pntd.0011132 (PMC9977039; doi:10.1371/journal.pntd.0011132)
Supplement: Attachment — Submitted filename: RESPONSE TO REVIEWERS_2.docx [file pntd.0011132.s006.docx]

**RESPONSE TO REVIEWERS**

Reviewer 3

**Detailed comments**

**Comment;** A minimum of 500 medical and paramedical female students from the three faculties Comment: please explain more in detail the differences between medical and paramedical students. Do they study together during the entire curriculum, did you consider to perform stratified analysis whit regard to this feature?

**Response:** Thank you very much for your comments, and mostly we explained the Nigerian context regarding the curriculum in the discussion section (Lines 398-403). We have now revised the methodology section to provide more clarity about the differences between medical and paramedical students; Both students category has a common curriculum in their first year, which changes in subsequent years. (Please see revised lines 195-197). However, we couldn’t perform a stratified analysis based on this feature, since we didn’t document this particular variable in the final data collection tool. (please see revised line 404-406).

Line 195-197: Students enrolled in the faculty of medicines and pharmacy were grouped as medical students, while those biomedical courses in the faculty of biosciences were grouped as paramedical students.

Line 404-406: Due to data limitations, this present study couldn’t explore other factors that are associated with knowledge among study participants, however our findings reiterate the need to consider investments and innovations in building capacity of medical/para-medical students and HCPs on FGS [36].

**Comment:** Line 194-197; A minimum of 500 medical and paramedical female students from the three faculties; biosciences, medical sciences and pharmacy, were targeted for recruitment. These students were approached through the headship of faculty-based student unions, and subsequently invited to participate in the study through personal invitation letters sent to their institutional email. Comment: From line 118 onwards you formulate why your research focus is female genital schistosomiasis however it is still not explained and not mentioned in the limitations why you would interview only female students. All medical students independently of their gender should be aware of all diseases independently of their gender. Or would you assume that only male students should be aware about prostate cancer? Please provide a rationale on your decision and mention this factor in your limitations.

**Response:** Thank you very much for this comment. We agree that awareness and knowledge about FGS should be of concern to both gender. However, we failed to include male students in our participants frame, and we consider that as a major limitation. The rationale for this decision as at the time this study was conducted was because our interest (in addition to profiling the awareness and knowledge) was to particularly explore experiences of females with probable symptoms of FGS when they sought medical care. This we thought would also provide supportive evidence to the assessment of expertise among health care professional. This rationale is flawed, and subsequent studies would consider inclusion of both gender in our design. We thank the reviewer for this comment, and we have included this as the major and first limitation.

Please see revised lines explaining the rationale for the inclusion of only females between lines 192-196

Also please see revised lines on limitation of study between lines 476-483

**Comment:** Line 302-305: Students in the 2nd (OR: 1.66, 95% CI: 1.0, 2.7), 4th (OR: 1.97, 95% CI: 1.2,3.2), and 6th (OR: 5.05, 95% CI: 0.4, 5.5) year have higher chances of been more informed about schistosomiasis than others. However, there were no significant association between year of study and knowledge about FGS (95% CI included 1 and p > 0.05). (Table 2). Comment: CIs among 2nd year students and 5th year students crosses 1. This indicates that the association is not significant. The statement that more advanced student would have a higher likelihood of being informed about FGS cannot be driven from your statistical calculations. I would formulate this more clearly. Also, the fact that only bivariate logistic regression was performed is a limitation. It is plausible that better knowledge might be dependent from other factors which were not assessed. I would be more cautious about my formulations.

**Response:** Thank you very much for your comments, we agree with you. But the significance is only considered when the range values of CI doesn’t include 1. So for students in the 2^nd^ year for FGS, you would observe that the lower bound is 1 and higher bound is 2.7. so the range of values in between doesn’t include 1, but rather started with 1. This was why the association was significant with a p-value of 0.04. We have further modified our text description as you have suggested.

Line 401-403: regression model found that medical and paramedical students in the second, fourth and sixth year of study have higher odds of been more informed about schistosomiasis, but we couldn’t find any association with FGS.

Line 404-406: Due to data limitations, this present study couldn’t explore other factors that are associated with knowledge among study participants, however our findings reiterate the need to consider investments and innovations in building capacity of medical/para-medical students and HCPs on FGS [36].

**Comment;** 351-356. Remove either information in the text or Table 5. information is redundant. 351-355. Of the 587 female MPMS, 83 presented a case of painful urination to the health care center and only 22 (26.5%) of them were asked if they had recent contact with fresh water bodies(p=0.458). Similarly, only 12 (38.7%) of the 31 students who presented hematuria were asked(p=0.035). In addition, only 44 (23.5%) of 187 who presented lower abdominal pain were asked (p=0.786), and only 31 (37.3%) of the 83 residents who presented symptoms of venereal diseases were asked (p=0.000) (Table 5). Comment: only in this paragraph it becomes clear that you have interviewed MPMS students in their function as professionals but also with regard to their experience as potential FGS patients-.I would consider removing this results section from your article it is an odd choice to ask students to slip into both roles and a very specific sample of patients which will no be representative for other groups. You have enough results anyhow. And this twist is very confusion also for an attentive reader. Otherwise please make this aspect clear in the methods section and evtl. In the title, please explain clearly in the methods why you choose to do so.

**Response:** Dear Reviewer, we would be glad if we could retain this table 5 and its corresponding text. This is primarily because this is the only table that justifies our inclusion of female students in the study.

Also, to provide more clarifications, we didn’t ask the students to act as professionals. What we did was to extract the profile of students who had experienced some of the probable symptoms of FGS and had visited the hospital for medical care. We extracted two separate variable from their datasheet; the symptoms experienced, and if the practitioner who provided care asked the student about a major risk factor (i.e., freshwater contact practice)

We have added more lines under the data management and analysis section to provide more clarifications

Please see line 248-253: Additionally, we extracted the anonymized dataset of students who had experienced some of the probable symptoms of FGS and had visited the hospital for medical care. We performed a cross-tabulation analysis on two variables; (i) the symptoms experienced, and (ii) if the practitioner who provided care asked about a major risk factor for FGS (i.e., freshwater contact practice). Chi-square test was also used to test for associations.

**Comment**: Table 2. Please consider moving the table into the attachment, very much information which give the impression stark results, while being statistically weak.

**Response:** Dear Reviewer, we would be glad if we could retain this table 2 within the manuscript and its corresponding text. This is not only because this was requested by you and one other reviewer. But also, because the recommendations from your previous comments (round 1) that led to this analysis, added more insights and clarity to the manuscript irrespective of the statistical weakness. Several masked datasets are now clearly shown. We thank you.
