# Supplementary material for: Profiling the knowledge of female medical/para-medical students, and expertise of health care professionals on female genital schistosomiasis in Anambra, South Eastern Nigeria
Source: PLoS Negl Trop Dis. 2023 Feb 16;17(2):e0011132. doi: 10.1371/journal.pntd.0011132 (PMC9977039; doi:10.1371/journal.pntd.0011132)
Supplement: Peer review history

# Peer review: Decision Letter 0

**DOI:** 10.1371/journal.pntd.0011132.r001

**Authors:** Georgios Pappas, Eva Clark

29 Nov 2022

Dear Dr Aribodor,

Thank you very much for submitting your manuscript "Profiling the knowledge of university female students and expertise of health care professionals in the management of female genital schistosomiasis in Anambra, South Eastern Nigeria." for consideration at PLOS Neglected Tropical Diseases. As with all papers reviewed by the journal, your manuscript was reviewed by members of the editorial board and by several independent reviewers. In light of the reviews (below this email), we would like to invite the resubmission of a significantly-revised version that takes into account the reviewers' comments. 

We cannot make any decision about publication until we have seen the revised manuscript and your response to the reviewers' comments. Your revised manuscript is also likely to be sent to reviewers for further evaluation.

When you are ready to resubmit, please upload the following:

[1] A letter containing a detailed list of your responses to the review comments and a description of the changes you have made in the manuscript. Please note while forming your response, if your article is accepted, you may have the opportunity to make the peer review history publicly available. The record will include editor decision letters (with reviews) and your responses to reviewer comments. If eligible, we will contact you to opt in or out.

[2] Two versions of the revised manuscript: one with either highlights or tracked changes denoting where the text has been changed; the other a clean version (uploaded as the manuscript file). 

Important additional instructions are given below your reviewer comments.

Please prepare and submit your revised manuscript within 60 days. If you anticipate any delay, please let us know the expected resubmission date by replying to this email. Please note that revised manuscripts received after the 60-day due date may require evaluation and peer review similar to newly submitted manuscripts.

Thank you again for your submission. We hope that our editorial process has been constructive so far, and we welcome your feedback at any time. Please don't hesitate to contact us if you have any questions or comments.

Sincerely,

Georgios Pappas

Academic Editor

PLOS Neglected Tropical Diseases

Eva Clark

Section Editor

PLOS Neglected Tropical Diseases

***********************

Please respond to reviewer comments

Reviewer's Responses to Questions

**Key Review Criteria Required for Acceptance?**

As you describe the new analyses required for acceptance, please consider the following:

**Methods**

-Are the objectives of the study clearly articulated with a clear testable hypothesis stated?

-Is the study design appropriate to address the stated objectives?

-Is the population clearly described and appropriate for the hypothesis being tested?

-Is the sample size sufficient to ensure adequate power to address the hypothesis being tested?

-Were correct statistical analysis used to support conclusions?

-Are there concerns about ethical or regulatory requirements being met?

Reviewer #1: 1. No, the objectives are not very clear and no clear testable hypothesis is stated. There is need for a clearer objective, and hypothesis stated

2. Yes, the study design is appropriate to address the stated objectives

3. No, the population is not clearly described at this time, and a bit confusing with sampling methods not described

4. Statistical methods used are okay

5. No ethical concerns, but the presentation of the ethical statements could be made clearer

Reviewer #2: Recommendation: Acceept

The objectives are clearly articulated, but the author needs to be more specific.

The study design is appropriate

The population is clearly defined, need to be specific for the students enrolled

Sample size is sufficient

Statistical analysis are correct

Ethics procedures were followed

Reviewer #3: Objectives of the study are clearly stated. Study design is appropriate. 

Study population is doubtful as only female students were included. Among the health workers also personell not qualified to provide FGS care were included such academic reserachers or lab personel. Please exclude those persons from your analysis.

Please provide information on the sample size and regarding power calculation. If not done please provide a reason for it.

Statistical analysis is appropriate for the reserach questions, futher it could be adresses which are the determinants of correct management of FGS using regression analysis.

--------------------

**Results**

-Does the analysis presented match the analysis plan?

-Are the results clearly and completely presented?

-Are the figures (Tables, Images) of sufficient quality for clarity?

Reviewer #1: 1. The analysis presented does not completely match the analysis plan, but can be reviewed. There is need for results to be reviewed and to represent study population description, and study objective questions as described in methods. 

2. The results are not very well clearly and completely presented

3. Yes, figures and tables are descriptive, though the variables presented in tables are not analyzed in the discussion.

Reviewer #2: The results match the analysis plan but the author needs to revise the text as recommended, in order to be clear. Table and figures are clear.

Reviewer #3: the results mach the analysis plan.

Results are clearly presented. Tables and images quality is ok.

--------------------

**Conclusions**

-Are the conclusions supported by the data presented?

-Are the limitations of analysis clearly described?

-Do the authors discuss how these data can be helpful to advance our understanding of the topic under study?

-Is public health relevance addressed?

Reviewer #1: (No Response)

Reviewer #2: Conclusion is supported by data presented.

Study limitations are clearly described.

The authors discussed how the data can be helpful in advancing our understanding of the impact of FGS knowledge gap

The public relevance is addressed

Reviewer #3: Conclusion support the data presention. However it should be stated more clearly that part of the mentioned diagnostic facilities are not suitable for the diagnosis of FGS which required the tools and the competences to perform colposcopy as well as the competence to diagnose pathognomic leasion, eventually with the help of a diagnostic atlas or with AI based App support. 

The limitiations of the analysis should adress the sample selection and stress further the distinction between the diagnosis of FGS and urinary schisto.

The autors adress how the data could advance the understanding of public health management.

--------------------

**Editorial and Data Presentation Modifications?**

Use this section for editorial suggestions as well as relatively minor modifications of existing data that would enhance clarity. If the only modifications needed are minor and/or editorial, you may wish to recommend “Minor Revision” or “Accept”. 

Reviewer #1: The 'Author summary' could be reviewed to present more background of the identified problem and contextualized within the selected study site.

Reviewer #2: Please see the attached pdf manuscript with comments.

Reviewer #3: see attached word document.

--------------------

**Summary and General Comments**

Use this section to provide overall comments, discuss strengths/weaknesses of the study, novelty, significance, general execution and scholarship. You may also include additional comments for the author, including concerns about dual publication, research ethics, or publication ethics. If requesting major revision, please articulate the new experiments that are needed.

Reviewer #1: This is an interesting topic, on the level of awareness and practices around schistosomiasis and female genital schistosomiasis among young female students and health practitioners in the South Eastern Nigeria. The authors have made a commendable attempt, but much still needs to be made clear, or presented differently, for a better understanding of their efforts. There is firstly an identified concern on the criteria and sampling strategy used, and how they arrived at the number of participants selected for interview; where this manuscript could benefit from a better presentation. Furthermore, the presentation of the results, and the translation of results to discussion, could benefit from more organization: here, the variables used and presented within the results such as gender, different age groups, years of practice and specialization of health practitioners questioned, could be considered further in analysis, to exploit better these results. There is need for a stronger discussion linked to the results presented here. 

This manuscript will benefit from a major review. 

--Abstract and through-out the manuscript.

It is understood from the concluding paragraph of the abstract (line 53), that the authors in referring to “female university students” may mean “medical and para-medical university students”. If this is the case, this manuscript may benefit in its over-all objective and precision if this is described as such early-on within the abstract and manuscript body.

--Line 118-124 and through-out the manuscript

Will benefit from references to support these statements

--Line 124-125

Once again please, are the authors referring to medical and para-medical students as supported on line 120-122?

--Line 127-129

How will this be useful? Please can the objective here be made clearer?

--Line 136-137

A word is lacking in the sentence.

--Line 137-138 

‘As applicable’ is unclear. Does this mean informed consent was gotten ‘where applicable’? Thus, when was it not applicable?

--Line 142-149, and further within the manuscript

There is a need for adequate referencing/citing within the manuscript

--I53-175

Was a sample size calculated or how were participants selected, and the number ‘587’ and ‘65’ (line 34) arrived at? Sampling methods and reasoning applied here need to be defined and explained 

--Line 153-154

Please the authors need to be precise on when this study was carried out

--Line 203,204, 207

What does ‘school environment’ refer to? Is this within curricula?

--Line 246-247

Question categories and reasoning behind this needs to be presented within the manuscript for clarity. For example, was ‘quarantine patients’ a category within the questioning, or was this gotten from respondents own understanding of the disease management? If it was a category, why?

--Line 249-251

Is this related to pediatric praziquantel? Or did researchers question respondents on praziquantel administration based on age? and not height/body weight? This is a bit confusing and might need to be clarified further. 

--Line 251

‘commodities’ here refer to medical equipment; medication?

--Line 320-323

‘Female residents’ mentioned here. Are these the inhabitants of the study site community or study participants other than the female university students and health workers? The authors will need to present these respondents within the participant selection and description paragraph, before results (seen on line 268 and elsewhere within the results section), before discussing them here. Participant categories have been declared early on within the manuscript as female students and health workers, with their Knowledge, Attitudes, and Practices around schistosomiasis and female genital schistosomiasis questioned. There is need to present other ‘female residents’ and the questioning on their experiences when presenting at health centers or hospitals when showing schistosomiasis/FGS symptoms within the methods part of the manuscript as well. 

Alternatively, if the description ‘female residents’ refers to ‘university students’ this has to be better written and consistent everywhere, to avoid misunderstanding, as well as the questioning on their hospital experiences brought out early-on when referring to the questions within the questionnaires administered.

Reviewer #2: The manuscript should be accepted, after revision as recommended on the attached pdf version. It provides an insight into the understanding of the impact of the knowledge gap among health care professionals and the need to address this gap during undergraduate study. The study findings provides opportunities for NTD programmes to advocate for more resources and integration with STIs and reproductive health programmes. More research in this area is still needed to compliment these findings.

Reviewer #3: the study gives an overview of knowledge of health care professionals and students in a region in Nigeria where FGS is endemic. 

It would be useful to stress more clearly how difficult it is to identify FGS and to adress the syndromic approach as a possible manner to reach more persons which might have FGS.

It would be useful to identify if the the mentioned university the curriculum includes FGS or schistosomiasis.

please refinde your study population as mentioned above.

--------------------

PLOS authors have the option to publish the peer review history of their article (what does this mean?). If published, this will include your full peer review and any attached files.

If you choose “no”, your identity will remain anonymous but your review may still be made public.

**Do you want your identity to be public for this peer review?** For information about this choice, including consent withdrawal, please see our Privacy Policy.

Reviewer #1: No

Reviewer #2: No

Reviewer #3: Yes: Angela Schuster

Figure Files:

While revising your submission, please upload your figure files to the Preflight Analysis and Conversion Engine (PACE) digital diagnostic tool, https://pacev2.apexcovantage.com. PACE helps ensure that figures meet PLOS requirements. To use PACE, you must first register as a user. Then, login and navigate to the UPLOAD tab, where you will find detailed instructions on how to use the tool. If you encounter any issues or have any questions when using PACE, please email us at figures@plos.org.

Data Requirements:

Please note that, as a condition of publication, PLOS' data policy requires that you make available all data used to draw the conclusions outlined in your manuscript. Data must be deposited in an appropriate repository, included within the body of the manuscript, or uploaded as supporting information. This includes all numerical values that were used to generate graphs, histograms etc.. For an example see here: http://www.plosbiology.org/article/info%3Adoi%2F10.1371%2Fjournal.pbio.1001908#s5.

Reproducibility:

To enhance the reproducibility of your results, we recommend that you deposit your laboratory protocols in protocols.io, where a protocol can be assigned its own identifier (DOI) such that it can be cited independently in the future. Additionally, PLOS ONE offers an option to publish peer-reviewed clinical study protocols. Read more information on sharing protocols at https://plos.org/protocols?utm_medium=editorial-email&utm_source=authorletters&utm_campaign=protocols

Attachment Submitted filename: Reviewer Comments.docx Click here for additional data file.

Attachment Submitted filename: PNTD-D-22-01326_reviewer.pdf Click here for additional data file.

Attachment Submitted filename: review_FGS.docx Click here for additional data file.

# Author comment: Author response to Decision Letter 0

**DOI:** 10.1371/journal.pntd.0011132.r002

5 Jan 2023

Attachment Submitted filename: Response to reviewers.docx Click here for additional data file.

# Peer review: Decision Letter 1

**DOI:** 10.1371/journal.pntd.0011132.r003

**Authors:** Georgios Pappas, Eva Clark

29 Jan 2023

Dear Dr Aribodor,

Thank you very much for submitting your manuscript "Profiling the knowledge of female medical/para-medical students, and expertise of health care professionals on female genital schistosomiasis in Anambra, South Eastern Nigeria." for consideration at PLOS Neglected Tropical Diseases. As with all papers reviewed by the journal, your manuscript was reviewed by members of the editorial board and by several independent reviewers. The reviewers appreciated the attention to an important topic. Based on the reviews, we are likely to accept this manuscript for publication, providing that you modify the manuscript according to the review recommendations. 

Some minor revisions are still needed. Please prepare and submit your revised manuscript within 30 days. If you anticipate any delay, please let us know the expected resubmission date by replying to this email. 

When you are ready to resubmit, please upload the following:

[1] A letter containing a detailed list of your responses to all review comments, and a description of the changes you have made in the manuscript. 

Please note while forming your response, if your article is accepted, you may have the opportunity to make the peer review history publicly available. The record will include editor decision letters (with reviews) and your responses to reviewer comments. If eligible, we will contact you to opt in or out

[2] Two versions of the revised manuscript: one with either highlights or tracked changes denoting where the text has been changed; the other a clean version (uploaded as the manuscript file).

Important additional instructions are given below your reviewer comments. 

Thank you again for your submission to our journal. We hope that our editorial process has been constructive so far, and we welcome your feedback at any time. Please don't hesitate to contact us if you have any questions or comments.

Sincerely,

Georgios Pappas

Academic Editor

PLOS Neglected Tropical Diseases

Eva Clark

Section Editor

PLOS Neglected Tropical Diseases

***********************

Reviewer's Responses to Questions

**Key Review Criteria Required for Acceptance?**

As you describe the new analyses required for acceptance, please consider the following:

**Methods**

-Are the objectives of the study clearly articulated with a clear testable hypothesis stated?

-Is the study design appropriate to address the stated objectives?

-Is the population clearly described and appropriate for the hypothesis being tested?

-Is the sample size sufficient to ensure adequate power to address the hypothesis being tested?

-Were correct statistical analysis used to support conclusions?

-Are there concerns about ethical or regulatory requirements being met?

Reviewer #1: (No Response)

Reviewer #3: please revise the maniscript and make more clear that a subset of medical an paramedical students were also interviewed in their function patients presentiing with FGS symptoms.

Please consider removing this aspect for more clarity.

Study design: Please clarify why only female students where interviewed this is still not clear to me

--------------------

**Results**

-Does the analysis presented match the analysis plan?

-Are the results clearly and completely presented?

-Are the figures (Tables, Images) of sufficient quality for clarity?

Reviewer #1: (No Response)

Reviewer #3: As stated above I would remove information on Med and paramed Students as patients. this aspect does not mach the title, the aim and the conclusion of your manuscript. 

Also the selection of potiential patients is very selective, potiential biases arise when administring a questionnaire which ask about knowledge and that ask about experienced symptoms....

Please put less enphasis on the results of the regression analysis, CI crosse 1 in many cases, CI are very large, only a bivariate regression was performed, I would be more cautious regarding the conclusions you draw from these results.

--------------------

**Conclusions**

-Are the conclusions supported by the data presented?

-Are the limitations of analysis clearly described?

-Do the authors discuss how these data can be helpful to advance our understanding of the topic under study?

-Is public health relevance addressed?

Reviewer #1: (No Response)

Reviewer #3: the conculsion section has improved considerably. PH relevance that understanding FGS has increased

--------------------

**Editorial and Data Presentation Modifications?**

Use this section for editorial suggestions as well as relatively minor modifications of existing data that would enhance clarity. If the only modifications needed are minor and/or editorial, you may wish to recommend “Minor Revision” or “Accept”. 

Reviewer #1: (No Response)

Reviewer #3: minor revisions

--------------------

**Summary and General Comments**

Use this section to provide overall comments, discuss strengths/weaknesses of the study, novelty, significance, general execution and scholarship. You may also include additional comments for the author, including concerns about dual publication, research ethics, or publication ethics. If requesting major revision, please articulate the new experiments that are needed.

Reviewer #1: (No Response)

Reviewer #3: 194

A minimum of 500 medical and paramedical female students from the three faculties

Comment: please explain more in detail the differences between medical and paramedical students. Do they study together during the entire curriculum, did you consider to perform stratified analysis whit regard to this feature? 

Line 194-197

A minimum of 500 medical and paramedical female students from the three faculties;biosciences, medical sciences and pharmacy, were targeted for recruitment. These students were approached through the headship of faculty-based student unions, and subsequently invited to participate in the study through personal invitation letters sent to their institutional email.

Comment: From line 118 onwards you formulate why your research focus is female genital schistosomiasis however it is still not explained and not mentioned in the limitations why you would interview only female students.

All medical students independently of their gender should be aware of all diseases independently of their gender. Or would you assume that only male students should be aware about prostate cancer? 

Please provide a rationale on your decision and mention this factor in your limitations. 

Line 302-305

Students in the 2nd (OR: 1.66, 95% CI: 1.0, 2.7), 4th (OR: 1.97, 95% CI: 1.2,3.2), and 6th (OR: 5.05, 95% CI: 0.4, 5.5) year have higher chances of been more informed about schistosomiasis than others. However, there were no significant association between

year of study and knowledge about FGS (95% CI included 1 and p > 0.05). (Table 2)

Comment: CIs among 2nd year students and 5th year students crosses 1. This indicates that the association is not significant. The statement that more advanced student would have a higher likelihood of being informed about FGS cannot be driven from your statistical calculations. 

I would formulate this more clearly. Also, the fact that only bivariate logistic regression was performed is a limitation. It is plausible that better knowledge might be dependent from other factors which were not assessed. I would be more cautious about my formulations.

351-356

Remove either information in the text or Table 5 information is redundant. 

351-355

Of the 587 female MPMS, 83 presented a case of painful urination to the health care center and only 22 (26.5%) of them were asked if they had recent contact with fresh water bodies(p=0.458). Similarly, only 12 (38.7%) of the 31 students who presented hematuria were asked(p=0.035). In addition, only 44 (23.5%) of 187 who presented lower abdominal pain wereasked (p=0.786), and only 31 (37.3%) of the 83 residents who presented symptoms of venereal

diseases were asked (p=0.000) (Table 5)

Comment: only in this paragraph it becomes clear that you have interviewed MPMS students in their function as professionals but also with regard to their experience as potential FGS patients-. 

I would consider removing this results section from your article it is an odd choice to ask students to slip into both roles and a very specific sample of patients which will no be representative for other groups. You have enough results anyhow. And this twist is very confusion also for an attentive reader. 

Otherwise please make this aspect clear in the methods section and evtl. In the title, please explain clearly in the methods why you choose to do so.

Table 2

Please consider moving the table into the attachment, very much information which give the impression stark results, while being statistically week.

--------------------

PLOS authors have the option to publish the peer review history of their article (what does this mean?). If published, this will include your full peer review and any attached files.

If you choose “no”, your identity will remain anonymous but your review may still be made public.

**Do you want your identity to be public for this peer review?** For information about this choice, including consent withdrawal, please see our Privacy Policy.

Reviewer #1: No

Reviewer #3: No

Figure Files:

While revising your submission, please upload your figure files to the Preflight Analysis and Conversion Engine (PACE) digital diagnostic tool, https://pacev2.apexcovantage.com. PACE helps ensure that figures meet PLOS requirements. To use PACE, you must first register as a user. Then, login and navigate to the UPLOAD tab, where you will find detailed instructions on how to use the tool. If you encounter any issues or have any questions when using PACE, please email us at figures@plos.org.

Data Requirements:

Please note that, as a condition of publication, PLOS' data policy requires that you make available all data used to draw the conclusions outlined in your manuscript. Data must be deposited in an appropriate repository, included within the body of the manuscript, or uploaded as supporting information. This includes all numerical values that were used to generate graphs, histograms etc.. For an example see here: http://www.plosbiology.org/article/info%3Adoi%2F10.1371%2Fjournal.pbio.1001908#s5.

Reproducibility:

To enhance the reproducibility of your results, we recommend that you deposit your laboratory protocols in protocols.io, where a protocol can be assigned its own identifier (DOI) such that it can be cited independently in the future. Additionally, PLOS ONE offers an option to publish peer-reviewed clinical study protocols. Read more information on sharing protocols at https://plos.org/protocols?utm_medium=editorial-email&utm_source=authorletters&utm_campaign=protocols

References

Please review your reference list to ensure that it is complete and correct. If you have cited papers that have been retracted, please include the rationale for doing so in the manuscript text, or remove these references and replace them with relevant current references. Any changes to the reference list should be mentioned in the rebuttal letter that accompanies your revised manuscript. If you need to cite a retracted article, indicate the article's retracted status in the References list and also include a citation and full reference for the retraction notice.

# Author comment: Author response to Decision Letter 1

**DOI:** 10.1371/journal.pntd.0011132.r004

31 Jan 2023

Attachment Submitted filename: RESPONSE TO REVIEWERS_2.docx Click here for additional data file.

# Editor report: Decision Letter 2

**DOI:** 10.1371/journal.pntd.0011132.r005

**Authors:** Georgios Pappas, Eva Clark

31 Jan 2023

Dear Aribodor,

We are pleased to inform you that your manuscript 'Profiling the knowledge of female medical/para-medical students, and expertise of health care professionals on female genital schistosomiasis in Anambra, South Eastern Nigeria.' has been provisionally accepted for publication in PLOS Neglected Tropical Diseases.

Before your manuscript can be formally accepted you will need to complete some formatting changes, which you will receive in a follow up email. A member of our team will be in touch with a set of requests.

Please note that your manuscript will not be scheduled for publication until you have made the required changes, so a swift response is appreciated.

IMPORTANT: The editorial review process is now complete. PLOS will only permit corrections to spelling, formatting or significant scientific errors from this point onwards. Requests for major changes, or any which affect the scientific understanding of your work, will cause delays to the publication date of your manuscript.

Should you, your institution's press office or the journal office choose to press release your paper, you will automatically be opted out of early publication. We ask that you notify us now if you or your institution is planning to press release the article. All press must be co-ordinated with PLOS.

Thank you again for supporting Open Access publishing; we are looking forward to publishing your work in PLOS Neglected Tropical Diseases.

Best regards,

Georgios Pappas

Academic Editor

PLOS Neglected Tropical Diseases

Eva Clark

Section Editor

PLOS Neglected Tropical Diseases

***********************************************************

# Editor report: Acceptance letter

**DOI:** 10.1371/journal.pntd.0011132.r006

**Authors:** Georgios Pappas, Eva Clark

13 Feb 2023

Dear Aribodor,

We are delighted to inform you that your manuscript, "Profiling the knowledge of female medical/para-medical students, and expertise of health care professionals on female genital schistosomiasis in Anambra, South Eastern Nigeria.," has been formally accepted for publication in PLOS Neglected Tropical Diseases.

We have now passed your article onto the PLOS Production Department who will complete the rest of the publication process. All authors will receive a confirmation email upon publication.

The corresponding author will soon be receiving a typeset proof for review, to ensure errors have not been introduced during production. Please review the PDF proof of your manuscript carefully, as this is the last chance to correct any scientific or type-setting errors. Please note that major changes, or those which affect the scientific understanding of the work, will likely cause delays to the publication date of your manuscript. Note: Proofs for Front Matter articles (Editorial, Viewpoint, Symposium, Review, etc...) are generated on a different schedule and may not be made available as quickly.

Soon after your final files are uploaded, the early version of your manuscript will be published online unless you opted out of this process. The date of the early version will be your article's publication date. The final article will be published to the same URL, and all versions of the paper will be accessible to readers.

Thank you again for supporting open-access publishing; we are looking forward to publishing your work in PLOS Neglected Tropical Diseases. 

Best regards,

Shaden Kamhawi

co-Editor-in-Chief

PLOS Neglected Tropical Diseases

Paul Brindley

co-Editor-in-Chief

PLOS Neglected Tropical Diseases
